# Effectiveness of *Streptococcus salivarius* probiotics on alleviating radiation-induced oral mucositis via inflammatory and microecological modulation: a prospective pragmatic interventional study in nasopharyngeal carcinoma

**DOI:** 10.3389/fimmu.2026.1745549

**Published:** 2026-03-03

**Authors:** Xiao-Tong Huang, Hui Feng, Ye Tan, Quan Wang, Jie-Mei Wei, Zhao-Dong Huang, Xin-Tong Wang, Hai-Jun Lu

**Affiliations:** 1 Department of Radiation Oncology, The Affiliated Hospital of Qingdao University, Qingdao, Shandong, China; 2 Medical College, Qingdao University, Qingdao, Shandong, China; 3 Department of Oncology, Qingdao Central Hospital of Rehabilitation University, Qingdao, Shandong, China; 4 Department of Neurology, Linyi Central Hospital, Linyi, Shandong, China; 5 Department of Invasive Technology, Linyi Central Hospital, Linyi, Shandong, China

**Keywords:** inflammation, IL-6, IL-8, microbiota, probiotics, radiation-induced oral mucositis, *Streptococcus salivarius* K12, *Streptococcus salivarius* M18

## Abstract

**Background:**

Radiation-induced oral mucositis (OM) is a prevalent and debilitating complication of head and neck radiotherapy, yet its severity varies markedly between patients. Emerging evidence suggests that this heterogeneity is influenced by the pre-existing oral microbiome and host inflammatory tone.

**Methods:**

This prospective, pragmatic interventional study grouped nasopharyngeal carcinoma (NPC) patients receiving chemoradiotherapy by probiotic exposure: no probiotic, *Streptococcus salivarius* K12 (SsK12), or *Streptococcus salivarius* M18 (SsM18). Weekly oral assessments were used to characterize the onset, duration, and severity of OM. Group-based trajectory modeling (GBTM) was used to identify OM trajectories. Univariate, multivariate, and mediation analyses were used to explore associated factors and potential relationships.

**Results:**

Among 69 evaluable patients, OM occurred in 95.7%, with severe OM (SOM) in 42.4%. Compared with non-probiotic group, SsM18 significantly delayed OM onset (*p* = 0.014), reduced SOM duration (*p* = 0.019), and shortened total OM duration (*p* = 0.031), outperforming SsK12. GBTM identified two distinct OM trajectories: ‘Rapid-Onset, Severe’ group and ‘Late-Onset, Mild’ group. Multivariate analysis revealed that elevated log-transformed Interleukin-6 levels (odds ratio [OR] = 4.20, *p* = 0.020), and high Beck Oral Assessment Scale (BOAS) score (OR = 3.06, *p* = 0.044) as independent predictors of ‘Rapid-Onset, Severe’ trajectory. The Teeth subdomain of BOAS was identified as an independent predictor for earlier OM onset (*p* = 0.042). Mediation analysis suggested that the association between a higher Teeth subdomain score and OM was partially mediated by IL-6 elevation (proportion mediated: 30–50%).

**Conclusions:**

Radiotherapy-induced OM was associated with baseline oral health and inflammatory status. SsM18 supplementation was associated with improved OM-related outcomes, suggesting a potential role for precision probiotic strategies.

**Clinical trial information:**

https://www.chictr.org.cn/, identifier ChiCTR2600118357.

## 1 Introduction

Radiation-induced oral mucositis (OM) remains a frequent and debilitating toxicity in head and neck radiotherapy (1, 2). It manifests clinically in over 90% of patients, with severe OM (SOM, grade 3–4) occurring in up to 70% and driving pain, dysphagia, weight loss, treatment interruptions, and inferior oncologic outcomes (3–7). The pathobiology of OM is classically described by Sonis’s five-stage model: initiation, signaling, amplification, ulceration, and healing (8, 9).

The inflammatory pathogenesis was already well stablished by recent research. Current evidence indicates that OM develops through a multi-phase process encompassing inflammatory/vascular injury, epithelial damage, ulceration with secondary microbial involvement, and subsequent healing (10, 11). Radiotherapy-induced DNA damage and reactive oxygen species (ROS) generation trigger early activation of nuclear factor-κB (NF-κB) and related signaling cascades, leading to the transcriptional upregulation of pro-inflammatory cytokines such as tumor necrosis factor-α (TNF-α), interleukin-1β (IL-1β), and interleukin-6 (IL-6) (9, 12). These mediators increase vascular permeability, promote recruitment of neutrophils and macrophages, and amplify tissue injury through matrix metalloproteinase activation and epithelial apoptosis (12–14). During the ulcerative phase, loss of epithelial integrity facilitates secondary bacterial and fungal colonization, whose endotoxins further stimulate cytokine release and sustain NF-κB-dependent inflammatory amplification, forming a self-perpetuating injury loop (8, 15, 16). Collectively, these studies establish inflammatory dysregulation and signal amplification as central drivers of OM pathobiology. However, inflammatory framework alone does not fully explain the marked inter-patient variability in OM severity under comparable radiotherapy regimens, indicating that some additional host-related factors may contribute to mucosal vulnerability.

Accumulating evidence suggests that the pre-existing oral microbiome is a critical determinant of mucosal resilience, which may partially account for this heterogeneity (17–20). Radiotherapy often induces oral dysbiosis characterized by an overgrowth of acidogenic taxa, decreased salivary pH, and impaired barrier function, which may be correlated with more severe and prolonged mucositis (21). For example, SOM has been reported to be associated with *Streptococcus mutans*, *Prevotella*, *Cardiobacterium*, *Granulicatella* and *Fusobacterium* (22, 23), while dysbiotic microbial communities may facilitate local acidification, biofilm formation, and inflammatory ligand release, thereby aggravating cytokine-mediated injury and delaying mucosal healing (24). Meta-analyses and randomized controlled trials demonstrate that probiotic supplementation mitigates radiotherapy-induced mucositis by restoring microbial balance and suppressing pro-inflammatory cytokine signaling (20, 25–27). These findings reposition OM as an ecologically mediated inflammatory disorder rather than a purely dose-dependent epithelial toxicity. Nevertheless, the interactions between microbial ecology and inflammatory responses in the development of radiation-induced OM remain incompletely characterized.

Importantly, current probiotic-based interventions for OM have largely focused on a limited number of strains and anatomical niches. The extensively studied oral probiotic *Streptococcus salivarius* K12 (SsK12), which predominantly colonizes the oropharyngeal mucosa, has been reported to exert anti-inflammatory effects, potentially through modulation of NF-κB signaling, and to confer clinical benefits during head and neck radiotherapy (28–30). However, the clinical benefits of SsK12 may derive more from alleviating pharyngeal inflammation and infection than from a direct intraoral effect on OM pathogenesis (31, 32). Emerging evidence indicates that local oral acidification is an important contributor to mucosal injury in OM (33, 34). The closely related strain *Streptococcus salivarius* M18 (SsM18), which shares a common genetic background with SsK12, possesses enzymatic properties relevant to modulation of local acidity (35–37). To date, however, SsM18 has not been evaluated in radiation-induced OM, nor has it been directly compared with SsK12 in patients with head and neck cancer.

Therefore, the present prospective pragmatic interventional study compares SsM18 and SsK12 in nasopharyngeal carcinoma (NPC) patients receiving concurrent chemoradiotherapy (CCRT). By integrating trajectory modeling, clinical predictor analysis, and cytokine-mediated pathway exploration, we aimed to characterize the associations between strain-specific probiotic exposure, inflammatory patterns, and the clinical course of radiation-induced OM, and to generate hypotheses regarding potential host–microbiome–inflammation interactions.

## 2 Methods

### 2.1 Study design and patients

This prospective pragmatic interventional study was conducted at the affiliated hospital of Qingdao university (Qingdao, China; approval no. QYFY-WZLL-30165). We searched patients pathologically diagnosed with NPC from March 1, 2023, to November 25, 2024, who planning to receive definitive CCRT, and a total of 108 patients attended.

**Figure 1:**
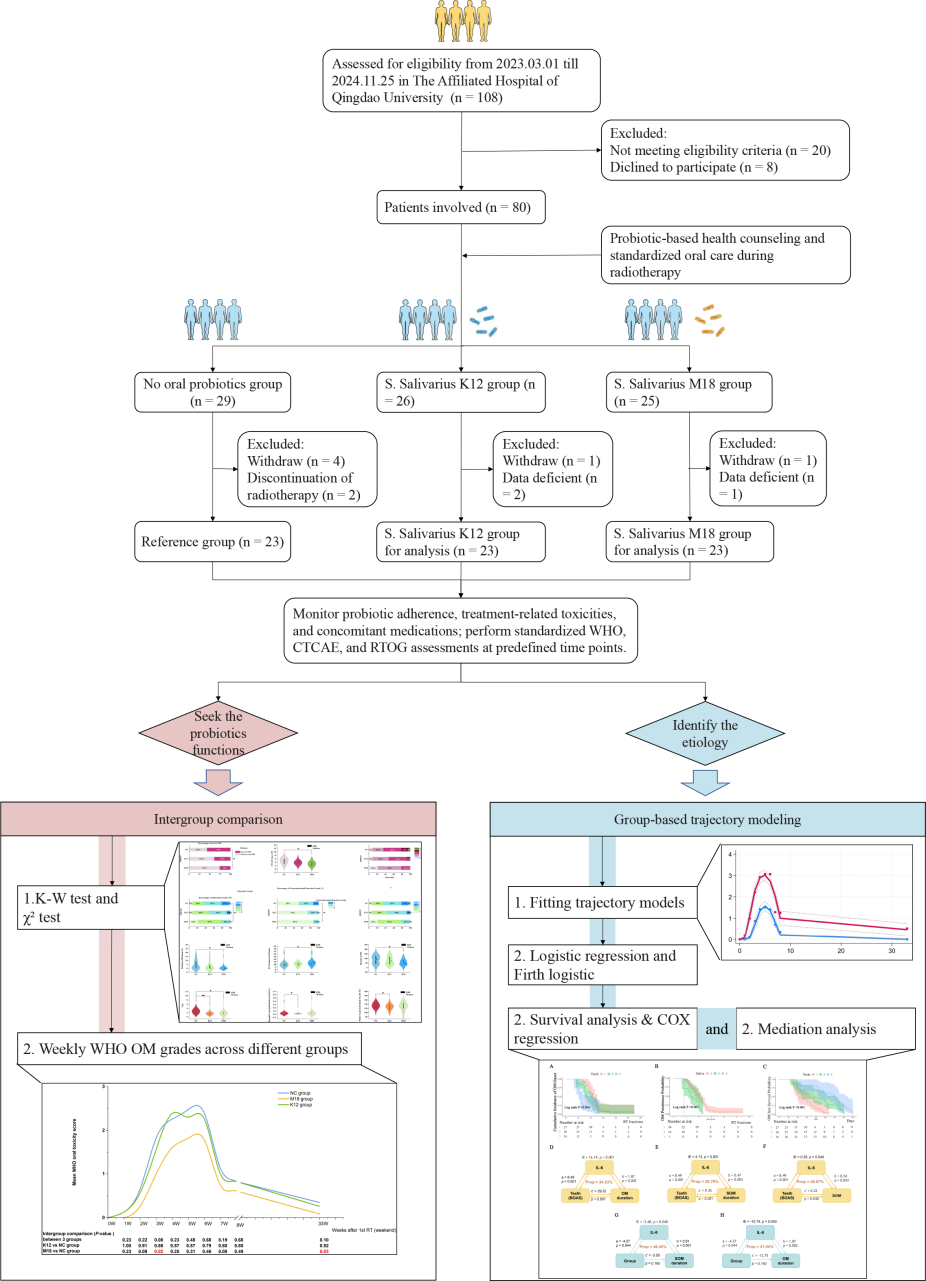
Flowchart illustrating the patient enrollment process and study design.

The inclusion criteria were as follows: (1) Newly diagnosed with histologically confirmed stage III-IVa NPC according to the 8th edition of the American Joint Committee on Cancer (AJCC) staging system; (2) No prior surgical intervention for nasopharyngeal tumor other than diagnostic biopsy; (3) Aged between 18 and 70 years; (4) Karnofsky Performance Status (KPS) score ≥ 70; (5) Provided written informed consent. The exclusion criteria included the following: (1) Clinically relevant organ dysfunction or comorbidities with the potential to compromise radiotherapy tolerance or bias outcome evaluation, including metabolic syndrome requiring intensive medical management; end-stage renal disease requiring hemodialysis; solid organ transplantation; diabetes mellitus with severe complications (diabetic ketoacidosis, hyperosmolar hyperglycemic state, or established end-organ damage); ischemic heart disease with unstable symptoms; active infectious diseases such as human immunodeficiency virus (HIV) infection or tuberculosis; clinically uncontrolled thyroid or other endocrine disorders; and autoimmune diseases requiring systemic treatment; (2) History of long-term use of immunosuppressive agents or corticosteroids (≥3 months); (3) Presence of another active malignancy; (4) Patients with severe pre-existing oral conditions (including active oral infection, uncontrolled periodontitis, ≥2 non–radiotherapy-related oral ulcers, oral abscesses, or other conditions requiring dental intervention); (5) Upper respiratory tract infection within the past 2 weeks or antibiotics used during follow-up; (6) History of head and neck radiation; (7) Presence of cognitive impairment or inability to cooperate with follow-up. (**Figure 1**).

### 2.2 Treatment plan

The target volume and radiotherapy plan for all enrolled patients were delineated and designed based on international consensus guidelines (38–40). The radiotherapy was delivered following the International Commission on Radiation Units and Measurements (ICRU) Reports No. 83 (41). The concurrent chemotherapy regimen consisted of cisplatin (100 mg/m^2^, administered every 3 weeks).

Before radiotherapy initiation, all patients completed comprehensive and professional oral pre-treatment by dentists to be appropriated for radiotherapy and received oral hygiene instructions with reference to multiple international guidelines on radiation. They maintained basic oral hygiene with Bass Method of toothbrushing, non-medicated mouthwashes, and a hospital-prepared Chinese herbal formula, Qingdi Mixture, which contained a blend of nine herbs (e.g., Rehmannia, Ophiopogon, Scutellaria) designed to provide anti-inflammatory, antioxidant, and immunomodulatory benefits against radiation-induced mucositis.

### 2.3 Grouping

Before the initiation of radiotherapy, all eligible patients received standardized oral-care education. To minimize physician- or patient-driven selection, eligible patients were sequentially assigned to one of three recommendation strategies in a 1:1:1 rotating sequence: no probiotic recommendation, recommendation of SsK12, or recommendation of SsM18, independent of clinical characteristics, contraindications, or patient preferences. Probiotic use was recommendation-based rather than mandated. Adherence was monitored prospectively throughout treatment. Patients who did not adhere to the assigned recommendation were excluded from the analysis cohort. The natural microbiota exposure group (reference group) served as a routine-care comparator, representing endogenous oral microbiota exposure in the absence of probiotic intervention, while The SsK12 and SsM18 groups represent routine-care-based probiotic supplementation strategies. This design represents a prospective pragmatic interventional study, and baseline characteristics were compared across groups to evaluate comparability.

The probiotic lozenges employed in this study contained SsK12 or SsM18, supplied as commercially available oral lozenges (Over-the-Counter dietary supplement). The products were manufactured by BLIS Technologies Ltd. (Dunedin, New Zealand) and marketed under the names BLIS K12™ and BLIS M18™. Each over-the-counter lozenge contained 2.5 × 10^9^ CFU of viable bacteria. From the start to the end of radiotherapy, patients were instructed to dissolve one lozenge in the oral cavity twice daily, followed by refraining from eating or drinking for at least 1 hour to promote colonization. The supplements were stored in a dry condition at 0–5 °C in a household refrigerator. Furthermore, the natural microbiota exposure group was instructed to rinse their mouths with normal saline twice a day. With routine supportive care, including Chinese patent medicines and analgesics, standardized across all groups per institutional protocols, probiotic exposure served as the main distinguishing intervention. However, patients who used antibiotics were withdrawn from the study.

### 2.4 Sample size estimation and statistical analysis

On the basis of our institutional data and previous reports, the average duration of OM in patients receiving head and neck chemoradiotherapy is approximately 90 days, and a reduction of about 30 days was considered clinically meaningful for patients receiving SsM18. Assuming a common standard deviation of 30 days (Cohen’s d ≈ 1.0), the required sample size for detecting this difference in a two-sample comparison of means (two-sided α = 0.05, 1 − β = 0.80) was calculated using the standard formula:


$$n=\frac{2{\left(z_{1-\alpha /2}+z_{1-\beta }\right)}^{2}\sigma^{2}}{{△}^{2}}$$

where *σ* is the common standard deviation and Δ is the expected mean difference. Substituting Z_1−α/2_ = 1.96, Z_1–β_ = 0.84, and Δ = 30 yields an estimated requirement of approximately 16 patients per group. As this exploratory cohort ultimately enrolled 23 patients per group, the achieved sample size exceeds this threshold and provides an estimated statistical power of approximately 90% to detect a 30-day difference in OM duration. Smaller or more modest differences may remain underpowered.

### 2.5 Assessment

Assessments were performed at baseline, weekly during radiotherapy, and at predefined post-treatment time points.

Prior to radiotherapy, all patients underwent assessment using the modified Beck Oral Assessment Scale (BOAS, **Supplementary Table 1**), by five domains: lips, gingiva, tongue, teeth, and saliva (42–44).

During radiotherapy, patients were followed weekly by two experienced radiation oncologists to document OM severity (WHO criteria), xerostomia, probiotic adherence, and concomitant medications, with post-radiotherapy follow-up at 2 weeks and 6 months for late OM degree (WHO criteria, **Supplementary Table 2**). SOM was defined as OM with a WHO grade 3–4 (5–7, 9, 45). OM and SOM duration were defined as the total number of consecutive days with WHO grade ≥1 and ≥3, respectively. For both, the start date was the first day the grade reached the respective threshold (≥1 for OM, ≥3 for SOM); the end date was the first day thereafter that the grade fell below that threshold without recurrence. The onset and resolution dates were determined based on patient-reported symptom timing and confirmed by trained investigators during radiotherapy or additional clinic visits. Both the calendar date and the corresponding radiotherapy fraction at onset and resolution were recorded. All durations were calculated based on continuous day counts rather than weekly intervals. Weekly grades were used exclusively for trajectory analysis and did not affect day-level duration calculations.

Blood samples were collected in the early morning at the time of the 15th radiotherapy fraction. After centrifugation at 1,400 × g for 15 minutes at 4 °C, serum levels of IL-6, Interleukin 8 (IL-8), Interferon-alpha (IFN-α), Interferon-gamma (IFN-γ), and TNF were quantified using ELISA kits (ThermoFisher Scientific, United States of America, USA), with absorbance measured at 450 nm on a Cytation multimode reader (BioTek, USA). Weekly routine blood tests, including complete blood count and albumin, were performed serially during treatment in hospital.

Acute radiotherapy-related toxicities were graded using Common Terminology Criteria for Adverse Events version 5.0 (CTCAE v5.0, **Supplementary Table 3**) at the 15th and 30th fractions and summarized for between-group comparisons. CTCAE v5.0 was also used to record treatment-emergent adverse events throughout radiotherapy for safety evaluation only including gastrointestinal disorders and hematologic toxicities (**Supplementary Table 4**). Hepatic and renal toxicities were defined by standard laboratory abnormalities (serum transaminases, serum creatinine, blood urea nitrogen, and estimated glomerular filtration rate). Late tissue toxicity was assessed using the Radiation Therapy Oncology Group (RTOG, **Supplementary Table 5**) criteria with post-radiotherapy follow-up at 6 months.

Additional parameters recorded throughout treatment included nutritional support, weight changes, radiotherapy interruptions, infections, and antibiotic use.

All assessments were independently performed and then jointly reviewed by two radiation oncologists, each with over five years of experience in head and neck radiotherapy–related toxicity assessment. Examiners underwent standardized training based on the established scoring scale and institutional guidelines prior to study initiation. Any discrepancies resolved through discussion to achieve a consensus grade.

### 2.6 Data collection

Of the 80 eligible patients enrolled, 69 completed the follow-up and were included in the analysis. Comprehensive baseline data were extracted from the Hospital Information System (HIS) and the Varian radiotherapy system. Data included demographic characteristics (sex, age, body mass index, BMI), medical history (diabetes, hypertension), lifestyle factors (heavy smoking, weekly standard drinks), and Nutritional Risk Screening 2002 (NRS2002) score (46) (**Supplementary Table 6**). Laboratory parameters consisted of complete blood cell counts (neutrophils, lymphocytes, monocytes, eosinophils, basophils, hemoglobin, platelet), albumin, and Epstein-Barr virus DNA load. Pathological characteristics included tumor differentiation, TNM stage, and clinical stage. Treatment-related variables covered the number of chemotherapy cycles, application of Tomotherapy, the dose to 50% of the oral cavity volume (ORC, D50%), and the mean dose to 30% of the bilateral parotid glands (PG, mean bilateral parotid dose, D30%). Heavy smoking was defined as a cumulative exposure of ≥ 10 pack-years (47–50). Alcohol consumption was assessed as the average number of standard drinks per week and modeled as a continuous variable, with one standard drink defined as 14 g of pure ethanol (51). Δ Albumin = Albumin (before radiotherapy) - Albumin (after radiotherapy). Radiotherapy interruption is defined as any unplanned suspension of treatment that lasts for 3 or more consecutive working days during the course of radiotherapy. Inflammatory-based indices were calculated as follows: neutrophil-to-lymphocyte ratio (NLR = absolute neutrophil count/absolute lymphocyte count) (52), platelet-to-lymphocyte ratio (PLR = platelet count/absolute lymphocyte count), lymphocyte-to-monocyte ratio (LMR = absolute lymphocyte count/absolute monocyte count), systemic immune-inflammation index (SII = platelets × neutrophils/lymphocytes) (53), systemic inflammation response index (SIRI = neutrophils × monocytes/lymphocytes) (54, 55). Nutritional indicator s such as the prognostic nutritional index (PNI) and body mass index (BMI) were calculated as follows: PNI = albumin + 5 × lymphocytes (56) and BMI = weight (kg)/height squared (m^2^).

### 2.7 Statistical analysis

Data were presented as mean (standard deviation, SD), median (interquartile range, IQR), or number (percentage, %) as appropriate. For comparisons of baseline characteristics across the three study groups (natural microbiota exposure, SsK12, and SsM18), the Kruskal–Wallis test was used for skewed continuous variables, one-way ANOVA for normally distributed continuous variables, and the Chi-square or Fisher’s exact test for categorical variables.

To evaluate the effects of probiotic supplementation on OM prevention and other treatment-related outcomes, follow-up data–including WHO oral toxicity score, patient quality of life scores, relevant clinical indicators, and medication use–were compared across the three groups using the same suite of non-parametric and parametric tests (Kruskal–Wallis test, one-way ANOVA, Chi-square test, or Fisher’s exact test). These analyses were conducted to demonstrate the efficacy of probiotics in mitigating radiotherapy-induced adverse events, particularly OM, and to investigate potential safety concerns. *Post-hoc* pairwise comparisons were performed using the Mann-Whitney U test for continuous variables and Fisher’s exact test for categorical variables to identify the specific sources of any significant overall differences.

Group-based trajectory modeling (GBTM) was employed to identify distinct longitudinal trajectories of OM severity during and after radiotherapy. We followed a two-step procedure based on Nagin’s work to determine the optimal trajectory model. The first step involved selecting the number of latent trajectories: starting with a single-group cubic model, we incrementally increased the number of groups and evaluated model fit using the Bayesian Information Criterion (BIC), average posterior probability of assignment (APPA > 0.70), odds of correct classification (OCC > 5.0), and a minimum of 5% of participants per group. The second step optimized the shape of trajectories by iteratively removing nonsignificant higher-order polynomial terms while retaining linear terms. Weekly OM assessments were prospectively collected, and patients with incomplete follow-up for OM trajectory or duration estimation were excluded from the corresponding analyses. No imputation or interpolation was performed.

Univariate logistic regression was used to identify clinical factors associated with assignment to an OM trajectory group. Variables with *p*< 0.1 and variance inflation factor (GVIF^(1/(2*Df)))< 5 in univariate analysis were included in a multivariable Firth penalized logistic regression model. This model aimed to identify independent predictors of trajectory group assignment, with the probiotic intervention variable excluded from this step. The Firth correction was applied both to reduce small-sample bias and to address potential separation due to sparse data. To reduce the risk of overfitting, the number of variables included in multivariable models was limited relative to the number of outcome events, and covariates were selected based on univariate screening and clinical relevance rather than automated selection procedures. Due to marked right-skewness, log-transformed values of IL-6 and weekly alcohol consumption (measured in standard drinks) were used in the multivariable models to enhance stability and limit the influence of extreme observations. Because BOAS was *a priori* selected as the primary oral health indicator, it was retained in multivariable models to ensure interpretability and consistency with the study design. The Teeth variable, being a sub-score of BOAS, was not entered into the same multivariable model to avoid conceptual overlap, structural multicollinearity, and potential overadjustment. Instead, its role was explored through stratified and interaction analyses within the BOAS framework.

Multiplicative interactions between different domains of BOAS (especially Teeth domain) and inflammatory biomarkers were evaluated to assess their joint effects on OM trajectories, which was done by including relevant cross-product terms in multivariable logistic regression models and assessing their significance with Wald tests. Specifically, we added cross-product terms (e.g., Teeth × IL-6) to the models. All interaction models were adjusted for potential covariates identified from univariate analyses (*p*< 0.10), including IL-8, weekly standard drinks, IFN-α, PNI, albumin, PLR, TNF, Nutritional Risk Screening (NRS) 2002 score, D50 of ORC, and the number of induction chemotherapy (IC) cycles. Classical additive interaction indices were not estimated because the outcome was a categorical trajectory rather than a time-to-event endpoint, and the limited sample size resulted in sparse data and separation across exposure combinations, precluding reliable estimation on the additive risk scale.

Cox proportional hazards analyses were conducted to further investigate factors associated with the timing of OM onset (the key difference between trajectories). For these analyses, the composite BOAS score was decomposed into its individual subscales. The model was adjusted for covariates that showed an association at *p*< 0.10 in univariable logistic regression. The number of radiotherapy fractions at OM onset was used as the time-to-event variable. The treatment variable was excluded from subsequent multivariable analyses to reduce confounding from intervention. Kaplan–Meier curves were generated to visualize the time to OM onset across different levels of key risk factors. Differences between groups were evaluated using the log-rank test. Kaplan-Meier curves were plotted according to the Teeth subdomain categories only for visual comparison (for descriptive purposes only).

Causal mediation analysis was performed based on multiple linear regression models to investigate the potential mechanisms through which clinical predictors influence OM-related outcomes. This analysis aimed to assess whether clinical predictors, such as probiotic supplementation (SsK12 or SsM18 groups versus the natural microbiota exposure reference group) and teeth status (BOAS dental domain scores 2 or 3 versus score 1), on OM duration and SOM risk were mediated by a panel of inflammatory biomarkers. The biomarkers examined included IL-6, IL-8, IFN-α, PNI. Mediation was considered significant if: (1) the exposure variable was significantly associated with the mediator; (2) the exposure variable was significantly associated with the outcome in a model without the mediator; and (3) the magnitude of the direct exposure–outcome association was attenuated after adjusting for the mediator in the model. The statistical significance of the indirect mediation effect was tested using the bias-corrected bootstrap method with 5,000 resamples to derive 95% confidence intervals (CIs). All mediation models were adjusted for weekly standard drinks, IFN-α, PNI, albumin, PLR, TNF, NRS 2002 score, D50 of ORC, and the number of IC cycles. The analyses relied on the standard assumptions of causal mediation analysis, including sequential ignorability (no unmeasured confounding). We tested for exposure–mediator interaction in preliminary models; as none were significant, final mediation models did not include interaction terms.

All regression, interaction and mediation models were adjusted for potential confounders. When IL-6 (or IL-8) was modeled as the mediator, the other cytokine (IL-8 or IL-6, respectively) was not adjusted for, to avoid overadjustment due to their biological coupling within the same inflammatory cascade. Other covariates were included for confounding control. Multicollinearity was assessed and confirmed absent (GVIF^(1/(2*Df))< 5). All tests were two-tailed with a significance level of *p*< 0.05. Statistical analyses were conducted using R (version 4.3.3) and Stata. R analyses were performed with the stats, car, MASS, logistf, survival, survminer, interactionR, and mediation packages. GBTM was performed using the traj package in Stata. All other visualizations were conducted using R version 4.3.3 or OriginLab 2024.

## 3 Result

### 3.1 Patient characteristics and follow-up completeness

Among the 108 patients initially screened, 80 met all inclusion and exclusion criteria and provided written informed consent. After follow-up, 23 patients from each group were included in the final analysis (**Figure 1**). The final cohort comprised predominantly male patients (69.6%), with a median age of 55 years. Common comorbidities and risk factors included hypertension (21.7%), diabetes mellitus (10.1%), and heavy smoking (26.1%). All patients were diagnosed with stage III–IV NPC, most with poorly differentiated histology (92.8%), and all received 2–4 cycles of induction chemotherapy prior to radiotherapy. Baseline characteristics were well balanced across the three groups (**Figure 1**; **Table 1**).

**Table 1:** Baseline Characteristics of 69 Patients before radiotherapy.

Variables	Total (n = 69)	Reference group (n = 23)	SsK12 group (n = 23)	SsM18 group (n = 23)	Statistic	*P*
BMI, Mean ± SD	24.27 ± 4.49	24.09 ± 3.56	24.87 ± 5.89	23.86 ± 3.78	F=0.32	0.730
Age, M (Q1, Q3)	55.00 (43.00, 62.00)	55.00 (45.00,59.00)	56.00 (35.00,62.00)	56.00 (46.00,63.00)	χ^2^ = 0.73#	0.694
Weekly standard drinks, M (Q1, Q3)	2.00 (0.00, 13.00)	2.00 (0.00,12.50)	2.00 (0.00,12.50)	5.00 (0.00,19.00)	χ^2^ = 0.15#	0.928
Neutrophil count, M (Q1, Q3)	3.55 (2.74, 4.34)	3.28 (1.83,4.96)	3.62 (3.03,4.53)	3.48 (2.74,3.97)	χ^2^ = 0.65#	0.723
Lymphocyte count, M (Q1, Q3)	1.82 (1.33, 2.18)	2.05 (1.37,2.50)	1.86 (1.29,1.95)	1.76 (1.29,1.98)	χ^2^ = 1.63#	0.442
Monocyte count, M (Q1, Q3)	0.52 (0.42, 0.62)	0.54 (0.43,0.61)	0.52 (0.41,0.57)	0.52 (0.39,0.64)	χ^2^ = 0.52#	0.772
Eosinophil count, M (Q1, Q3)	0.07 (0.04, 0.13)	0.07 (0.04,0.10)	0.07 (0.03,0.11)	0.08 (0.05,0.22)	χ^2^ = 2.41#	0.300
Basophil count, M (Q1, Q3)	0.03 (0.02, 0.05)	0.03 (0.02,0.05)	0.03 (0.02,0.04)	0.04 (0.03,0.06)	χ^2^ = 3.49#	0.175
Hemoglobin count, M (Q1, Q3)	140.00 (132.00, 151.00)	139.00 (129.00,149.50)	139.00 (128.00,148.00)	142.00 (137.00,155.00)	χ^2^ = 3.69#	0.158
Platelet count, M (Q1, Q3)	231.00 (198.00, 261.00)	231.00 (203.00,256.00)	237.00 (193.50,281.00)	227.00 (193.50,248.00)	χ^2^ = 0.61#	0.736
Albumin count, M (Q1, Q3)	43.70 (41.60, 45.60)	44.00 (42.00,45.50)	43.80 (40.85,45.55)	43.60 (41.75,45.15)	χ^2^ = 0.41#	0.815
EBV DNA copy number, M (Q1, Q3)	544.50 (0.00, 2710.00)	753.60 (0.00,2739.50)	544.50 (62.50,4875.00)	516.50 (0.00,1817.50)	χ^2^ = 0.37#	0.831
D50 of ORC, M (Q1, Q3)	16.10 (5.40, 23.33)	14.00 (5.95,22.77)	15.12 (6.70,21.12)	16.70 (5.05,28.75)	χ^2^ = 0.60#	0.739
D30 of PGs, M (Q1, Q3)	62.91 (48.10, 73.75)	64.61 (47.92,77.58)	63.12 (49.10,70.00)	53.00 (47.73,75.53)	χ^2^ = 0.97#	0.616
SCC, M (Q1, Q3)	1.41 (1.04, 1.93)	1.39 (1.11,1.75)	1.22 (0.97,1.80)	1.59 (1.04,1.98)	χ^2^ = 0.71#	0.700
CEA, M (Q1, Q3)	1.57 (1.10, 2.37)	1.42 (0.95,2.21)	1.23 (0.95,2.06)	2.30 (1.32,3.29)	χ^2^ = 5.55#	0.062
CYFRA21-1, M (Q1, Q3)	2.80 (2.14, 5.00)	2.67 (2.23,5.19)	3.35 (2.18,7.36)	2.87 (1.92,4.50)	χ^2^ = 1.22#	0.544
BOAS, M (Q1, Q3)	6.00 (5.00, 7.00)	6.00 (5.50,7.00)	6.00 (5.50,7.00)	6.00 (5.00,7.00)	χ^2^ = 0.78#	0.677
Male, n (%)					χ^2^ = 2.88	0.238
0	21 (30.43)	10 (43.48)	6 (26.09)	5 (21.74)		
1	48 (69.57)	13 (56.52)	17 (73.91)	18 (78.26)		
Diabetes, n (%)					–	1.000
0	62 (89.86)	21 (91.30)	20 (86.96)	21 (91.30)		
1	7 (10.14)	2 (8.70)	3 (13.04)	2 (8.70)		
Hypertension, n (%)					χ^2^ = 0.51	0.774
0	54 (78.26)	19 (82.61)	18 (78.26)	17 (73.91)		
1	15 (21.74)	4 (17.39)	5 (21.74)	6 (26.09)		
Heavy smoking, n (%)					χ^2^ = 1.80	0.406
0	51 (73.91)	19 (82.61)	17 (73.91)	15 (65.22)		
1	18 (26.09)	4 (17.39)	6 (26.09)	8 (34.78)		
NRS 2002 score, n (%)					–	0.453
1	35 (50.72)	10 (43.48)	13 (56.52)	12 (52.17)		
2	23 (33.33)	8 (34.78)	9 (39.13)	6 (26.09)		
3	7 (10.14)	4 (17.39)	0 (0.00)	3 (13.04)		
4	4 (5.80)	1 (4.35)	1 (4.35)	2 (8.70)		
Differentiation degree, n (%)					–	0.238
1	64 (92.75)	21 (91.30)	23 (100.00)	20 (86.96)		
2	3 (4.35)	2 (8.70)	0 (0.00)	1 (4.35)		
3	2 (2.90)	0 (0.00)	0 (0.00)	2 (8.70)		
T stages, n (%)					–	0.905
1	7 (10.14)	2 (8.70)	2 (8.70)	3 (13.04)		
2	27 (39.13)	11 (47.83)	7 (30.43)	9 (39.13)		
3	20 (28.99)	5 (21.74)	8 (34.78)	7 (30.43)		
4	15 (21.74)	5 (21.74)	6 (26.09)	4 (17.39)		
N stages, n (%)					–	0.361
0	6 (8.70)	2 (8.70)	3 (13.04)	1 (4.35)		
1	7 (10.14)	0 (0.00)	4 (17.39)	3 (13.04)		
2	34 (49.28)	14 (60.87)	10 (43.48)	10 (43.48)		
3	22 (31.88)	7 (30.43)	6 (26.09)	9 (39.13)		
Clinical stage, n (%)					–	0.943
3	38 (55.07)	13 (56.52)	13 (56.52)	12 (52.17)		
4	31 (44.93)	10 (43.48)	10 (43.48)	11 (47.83)		
Number of IC cycles, n (%)					–	0.148
2	54 (78.26)	19 (82.61)	16 (69.57)	19 (82.61)		
3	13 (18.84)	4 (17.39)	7 (30.43)	2 (8.70)		
4	2 (2.90)	0 (0.00)	0 (0.00)	2 (8.70)		
Tomotherapy, n (%)					χ^2^ = 0.12	0.942
0	41 (59.42)	14 (60.87)	13 (56.52)	14 (60.87)		
1	28 (40.58)	9 (39.13)	10 (43.48)	9 (39.13)		
Lips (BOAS), n (%)					–	1.000
1	64 (92.75)	22 (95.65)	21 (91.30)	21 (91.30)		
2	5 (7.25)	1 (4.35)	2 (8.70)	2 (8.70)		
Gingiva (BOAS), n (%)					–	0.512
1	61 (88.41)	19 (82.61)	20 (86.96)	22 (95.65)		
2	8 (11.59)	4 (17.39)	3 (13.04)	1 (4.35)		
Tongue (BOAS), n (%)					–	1.000
1	68 (98.55)	23 (100.00)	22 (95.65)	23 (100.00)		
2	1 (1.45)	0 (0.00)	1 (4.35)	0 (0.00)		
Teeth (BOAS), n (%)					χ^2^ = 0.65	0.957
1	27 (39.13)	9 (39.13)	8 (34.78)	10 (43.48)		
2	26 (37.68)	8 (34.78)	10 (43.48)	8 (34.78)		
3	16 (23.19)	6 (26.09)	5 (21.74)	5 (21.74)		
Saliva (BOAS), n (%)					–	0.933
1	54 (78.26)	18 (78.26)	17 (73.91)	19 (82.61)		
2	14 (20.29)	5 (21.74)	5 (21.74)	4 (17.39)		
3	1 (1.45)	0 (0.00)	1 (4.35)	0 (0.00)		

Lips, gingiva, tongue, teeth, and saliva are baseline oral subscale scores of BOAS. F, ANOVA; #, Kruskal-Wallis test; χ^2^, Chi-square test; -, Fisher’s exact test; BMI, Body mass index; EBV, Epstein-Barr virus; ORC, Oral rinsing count; SCC, Squamous cell carcinoma antigen; CEA, Carcinoembryonic antigen; CYFRA21-1, Cytokeratin 19 fragment antigen 21-1; BOAS, Beck Oral Assessment Scale; IC, Induction chemotherapy.

The overall incidence of OM was 95.7% (66/69). SOM occurred in 42.4% of patients. OM onset occurred at a median of 11th radiotherapy fraction, and the median duration was 71 days. OM increased rapidly during radiotherapy, with 87.0% occurring within the first three weeks of treatment, followed by prompt resolution after radiotherapy completion. All cases of SOM resolved within one week after radiotherapy, and 76.8% achieved complete mucosal recovery at the six-month follow-up. Radiotherapy adherence was high, with 91.7% of patients completing treatment as scheduled. Brief interruptions (<3 days) were rare and did not materially affect the analysis.

### 3.2 Between-group comparisons of clinical toxicity and supportive care (descriptive analysis)

#### 3.2.1 Weekly distributions of WHO OM grades and overall group differences

**Figure 2:**
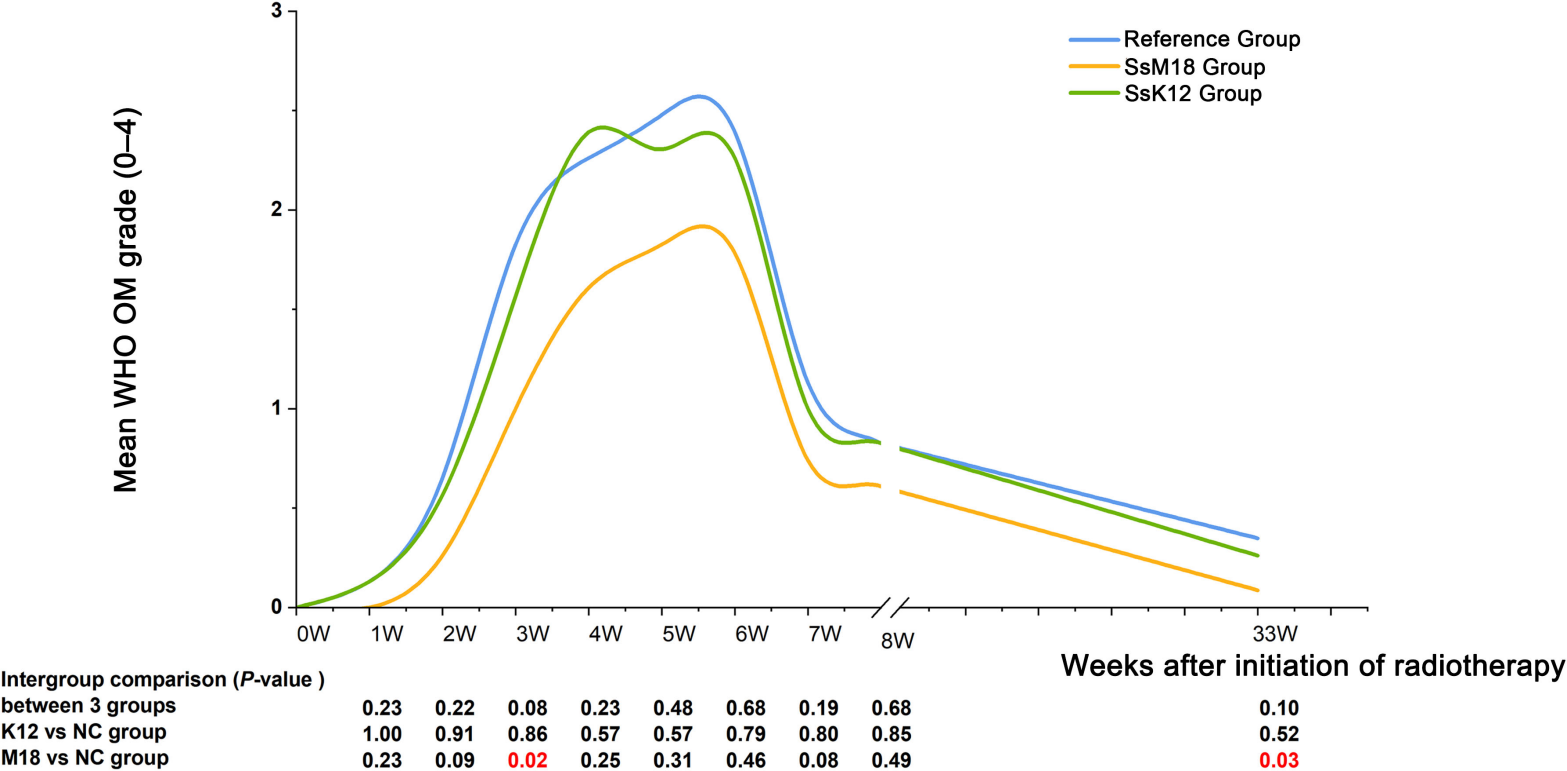
Weekly trends in OM severity during and after radiotherapy. Mean WHO OM grades are shown for each group at each scheduled weekly assessment. Curves are displayed as smoothed lines for visualization only. Intergroup P values shown below the figure were derived from comparisons of the weekly distributions of WHO OM grades among groups, using the χ^2^ test or Fisher’s exact test. Analyses were based on WHO grade distributions rather than the patient number with SOM.

**Figure 2** presents the observed weekly mean WHO OM grades in each group. The SsM18 group consistently exhibited lower WHO scores than the reference group. A statistically significant difference was observed in the 3rd week of radiotherapy (after 15 fractions; *p* = 0.022). Better mucosal recovery was observed in the SsM18 group at the six-month post-radiotherapy follow-up (*p* = 0.032). (**Supplementary Table 7**).

**Figure 3:**
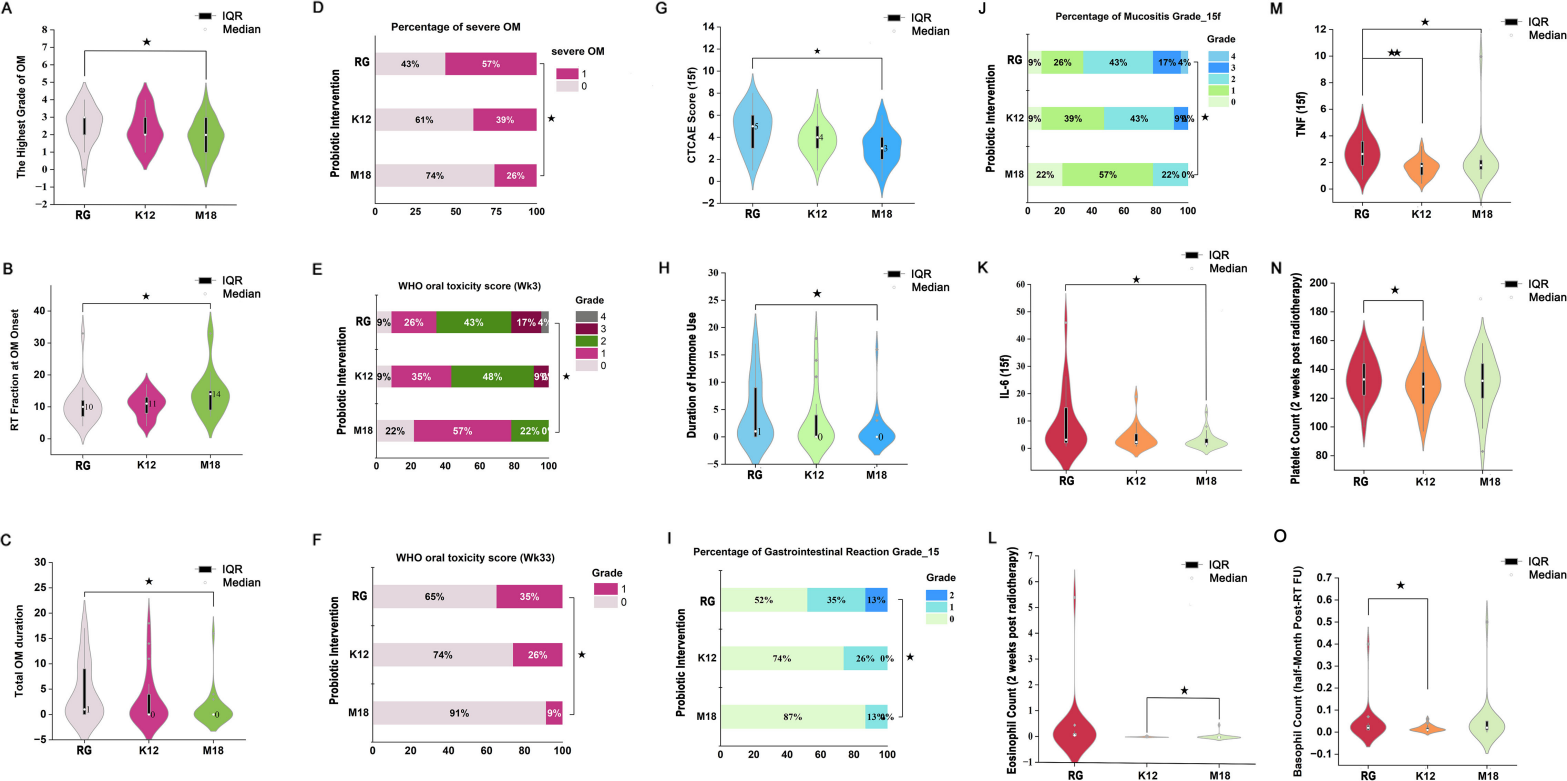
Effects of probiotic interventions on oral mucositis, treatment tolerance, and hematological parameters. Among the three groups: **(A)** Highest grade of OM; **(B)** Radiation fractions at OM onset; **(C)** Total duration of OM; **(D)** Incidence of SOM (≥grade 3); **(E)** Distribution of WHO OM grades in Week 3 of radiotherapy; **(F)** Distribution of WHO OM grades in Week 33 (post-radiotherapy follow-up at 6 months); **(G)** CTCAE total score at the 15^th^ fractions; **(H)** Duration of hormone use during radiotherapy; **(I)** Distribution of gastrointestinal reaction grades during the first 15 fractions (CTCAE v5.0); **(J)** Distribution of mucositis grades at the 15th fraction (CTCAE v5.0); **(K)** Serum IL-6 levels at 15th fraction; **(L)** Eosinophil count at 2 weeks after radiotherapy completion; **(M)** Serum TNF levels at 15th fraction; **(N)** Platelet count at half-month after radiotherapy completion; **(O)** Basophil count at half-month after radiotherapy completion. Violin plots show the distribution of data with the median and the interquartile range (IQR) indicated. Stacked bar charts present the proportion of patients in each grade category. RG: Reference group; K12: SsK12 group; M18: SsM18 group. Statistical significance is indicated by asterisks (★:P< 0.05, ★★:P< 0.01).

At the level of specific temporal outcomes, patients receiving SsM18 experienced a significantly delayed OM onset (median: 14 vs. 10 fractions, *p* = 0.014), a lower peak WHO grade (*p* = 0.039), and shorter durations of both OM (median: 47 vs. 93 days, *p* = 0.031) and SOM (*p* = 0.019). In contrast, the SsK12 group showed no statistically significant differences compared with reference group. (**Figure 3**; **Supplementary Table 8**).

#### 3.2.2 Clinical symptom relief and inflammatory modulation in the SsM18 group

The clinical benefits of OM mitigation were most apparent in the SsM18 group. At mid-radiotherapy (15 fractions), patients in the SsM18 group experienced significantly lower global CTCAE scores compared with the reference group (*p* = 0.009), driven by reduced mucosal reaction (*p* = 0.022) and dysphagia (*p* = 0.024). Notably, SsM18 also demonstrated superior efficacy compared with SsK12, achieving a significantly lower global CTCAE score at the same time point (*p* = 0.038). (**Supplementary Table 8**).

This symptomatic benefit was accompanied by a reduced need for intravenous hormones for symptom control in the SsM18 group compared with the reference group (p = 0.010). During radiotherapy, both probiotic groups exhibited significantly lower TNF levels than the reference group (SsK12: *p*< 0.001; SsM18: *p* = 0.005). IL-6 levels were also significantly lower in the SsM18 group compared with the reference group (*p* = 0.020). No significant differences in xerostomia severity were observed among the three groups during treatment or follow-up. (**Figure 3**; **Supplementary Table 8**).

#### 3.2.3 Safety and adverse events

No severe adverse events (SAEs) related to microbiota exposure were observed. The adverse events recorded during treatment were consistent with the profile of common radiotherapy-related toxicities. Among the 69 patients, myelosuppression occurred in 26 (37.7%), gastrointestinal adverse events in 20 (29.0%), and hepatic toxicity in 2 (2.9%). No cases of renal impairment were observed. Specifically, the incidence of gastrointestinal reactions was significantly higher in the natural microbiota exposure group (47.82%) compared to the probiotic groups (SsK12: 26.09%; SsM18: 13.04%; *p* = 0.046). In contrast, the incidence of myelosuppression and hepatic toxicity did not differ significantly among the three groups. (**Figure 3**; **Supplementary Table 8**).

### 3.3 Trajectory analysis of OM severity and associated factors

#### 3.3.1 Trajectory groups and proportions

**Figure 4:**
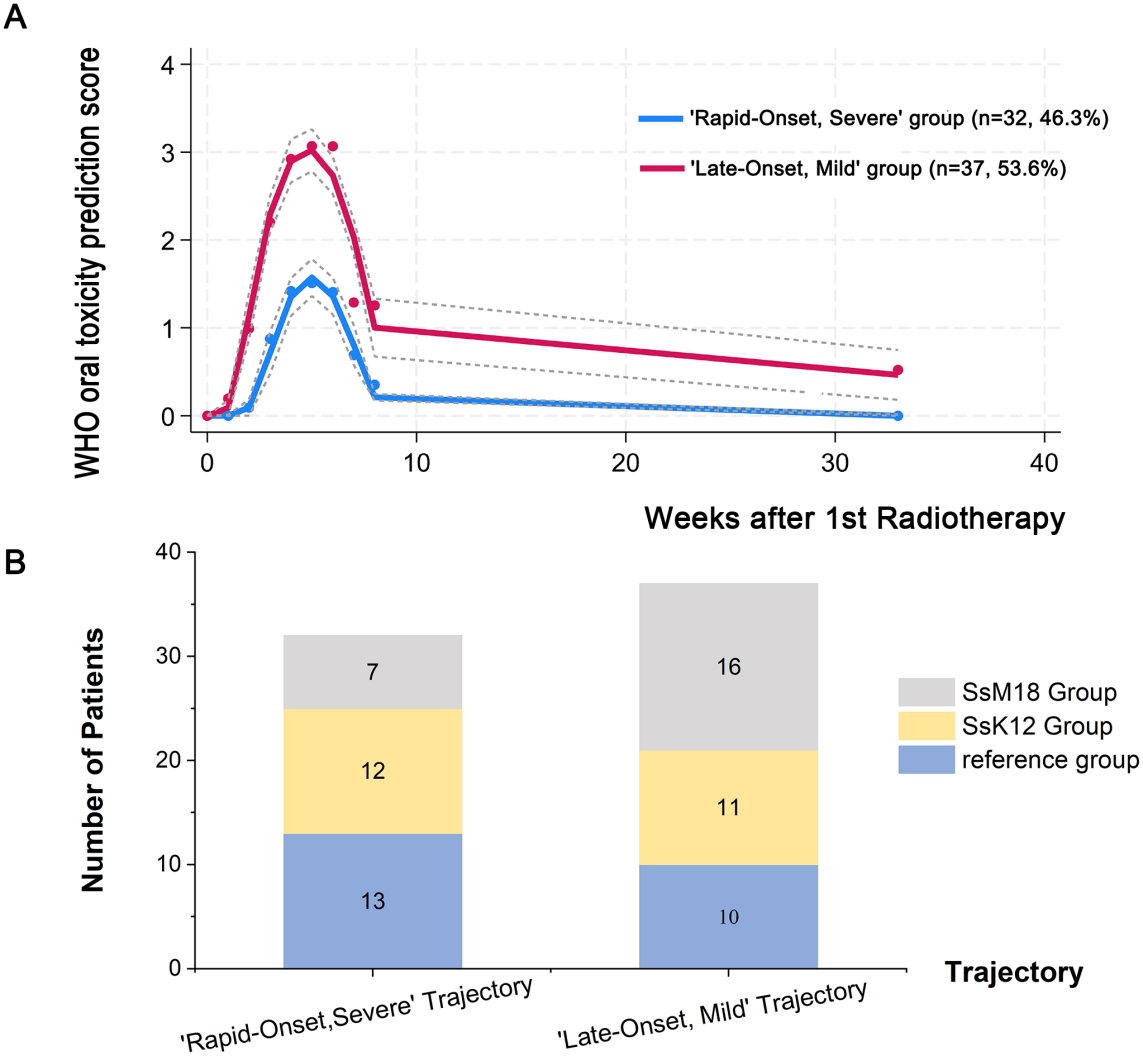
Trajectories of the WHO oral toxicity prediction score. **(A)** Solid lines (red: ‘Rapid-Onset, Severe’ group, blue: ‘Late-Onset, Mild’ group) indicate estimated values. The x-axis represents the number of weeks after the start of radiotherapy. **(B)** Distribution of probiotic exposure across OM trajectory groups.

To further explore heterogeneity in OM progression, GBTM was fitted to weekly WHO OM grades. Model fit indices identified a two-trajectory cubic model as optimal (BIC = −648.21; average posterior probability of assignment = 0.92), revealing two distinct OM progression phenotypes (**Figure 4A**; **Supplementary Table 9**).

The ‘Rapid-Onset, Severe’ trajectory (n = 32, 46.3%) was characterized by an early and steep increase in OM severity, reaching WHO grade ≥2 by week 4 (approximately 20 fractions), followed by a rapid post-radiotherapy decline but incomplete long-term resolution. In contrast, the ‘Late-Onset, Mild’ trajectory (n = 37, 53.6%) exhibited delayed onset, milder peak severity (never exceeding grade 2), and near-complete resolution by week 33. (**Figure 4A**; **Supplementary Table 9**).

As shown in **Figure 4B**, patients, treated with SsM18 were less likely to follow the ‘Rapid-Onset, Severe’ trajectory and more likely to belong to the ‘Late-Onset, Mild’ trajectory compared with the reference group.

#### 3.3.2 Factors associated with OM trajectories and interaction analysis

Univariate logistic regression identified several factors associated with the OM trajectory, including BOAS score, IL-6, IL-8, IFN-α, PNI, weekly standard drinks, and albumin (**Supplementary Table 10**). Despite not reaching statistical significance (*p* = 0.078), SsM18 supplementation showed a strong trend toward reducing the risk of belonging to the ‘Rapid-Onset, Severe’ trajectory (odds ratio [OR] = 0.34, 95% CI: 0.10–1.10). (**Supplementary Table 10**).

A multivariable Firth penalized logistic regression model identified elevated log-transformed IL-6 level (OR = 4.20, 95% CI: 1.27–13.86, *p* = 0.020), and high BOAS score (OR = 3.06, 95% CI: 1.11–8.44, *p* = 0.044) were identified as significant independent predictors of the ‘Rapid-Onset, Severe’ trajectory. (**Supplementary Tables 11**, **12**).

Analysis of multiplicative interaction found no significant effect modification (interaction *p* = 0.61).

#### 3.3.3 Sub-analysis of BOAS subcomponents associated with OM onset timing

**Figure 5:**
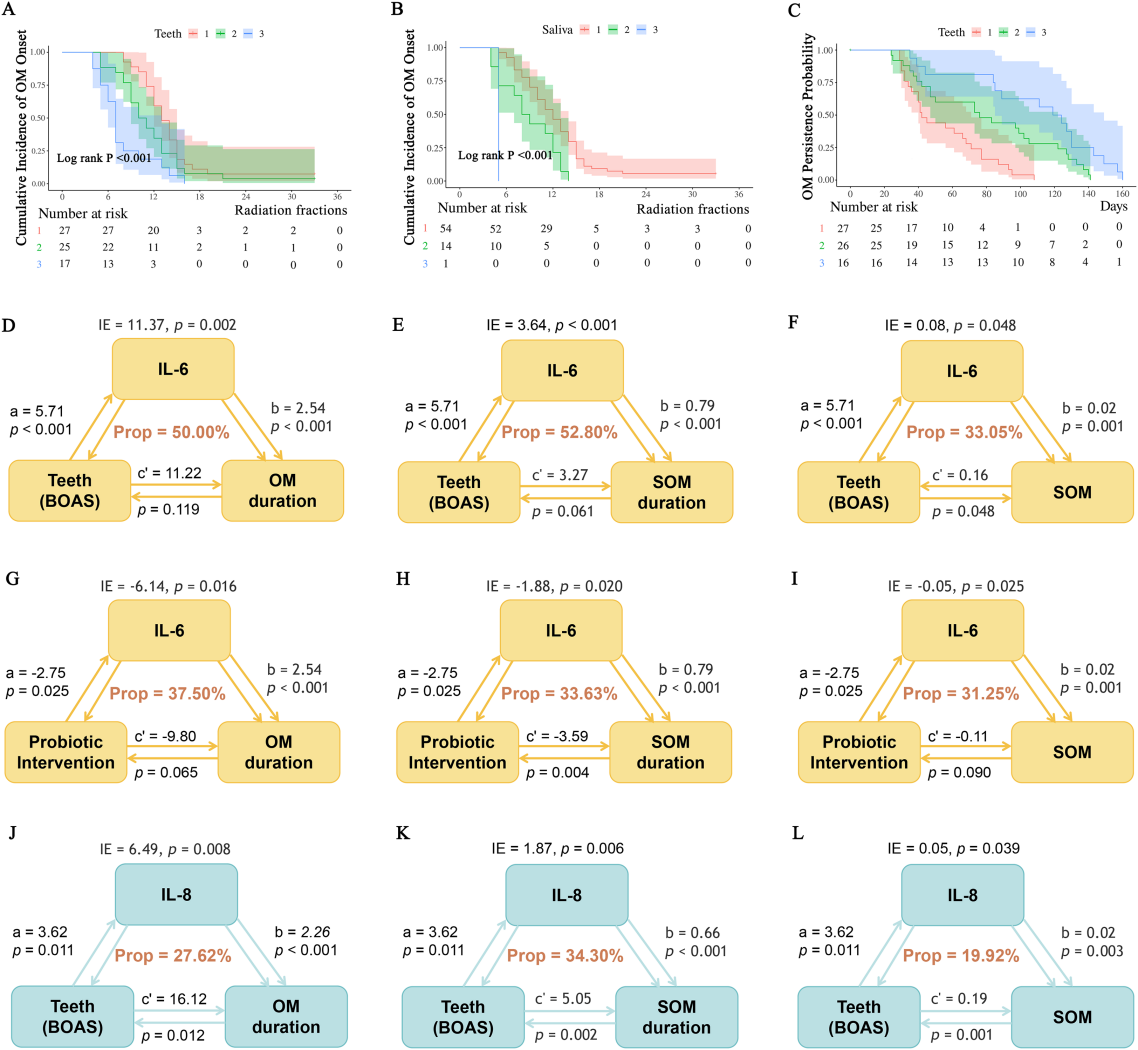
Host–microbiome–inflammation interactions underlying radiation-induced OM: survival and mediation analyses (exploratory). **(A, B)** Kaplan–Meier curves and log-rank tests for time to OM onset, stratified by BOAS–Teeth domain **(A)** and BOAS–Saliva domain **(B)**. **(C)** Kaplan–Meier curves illustrating OM duration by BOAS–Teeth subdomain (for descriptive visualization only). In **(A–C)**, scores 1, 2, and 3 denote BOAS subdomain score, with higher scores indicating poorer oral health status. **(D–L)** Exploratory mediation models linking oral health/probiotic exposure, inflammatory biomarkers, and OM-related outcomes (hypothesis-generating). Probiotic intervention indicates treatment assignment to reference group, SsK12, or SsM18 group. Path coefficients are shown for: a (exposure → mediator), b (mediator → outcome adjusted for exposure), and c′ (direct exposure → outcome adjusted for mediator). IE denotes the indirect effect (a×b). Prop indicates the proportion of the total association statistically mediated (indirect/total), shown as a percentage.

Univariable Cox regression identified several factors significantly associated with the timing of OM onset (measured in radiotherapy fractions), including SsM18 supplementation, a high NRS 2002 score, higher scores on the Tongue, Teeth, and Saliva subdomains of the BOAS, and elevated IL-6 and IL-8 levels (**Supplementary Table 13**). Multivariable Cox regression analysis demonstrated that the higher scores of Teeth subdomain remained independently associated with earlier OM onset (score 3 vs 1, hazard ratio [HR] = 2.50, 95% CI: 1.03–6.03, *p* = 0.042, **Supplementary Table 14**). Kaplan–Meier curves visualizing the time to OM onset are presented in **Figures 5A, B**, with log-rank tests showing significant association across Teeth and Saliva subdomains (both *p*< 0.001). **Figure 5C** depicts differences in OM duration by Teeth subdomain using Kaplan–Meier curves, but the log-rank statistic was not interpreted for inferential purposes.

#### 3.3.4 Inflammatory biomarkers as potential mediators of OM severity and duration

Mediation analyses were performed to assess whether inflammatory biomarkers explained the associations of dental status and probiotic supplementation with OM outcomes. IL-6 emerged as a consistent and substantial mediator. It accounted for approximately 30–50% of the associations between BOAS score (Teeth domain) on prolonged OM duration, SOM duration, and higher SOM risk, and similarly mediated the favorable associations of probiotic supplementation on OM severity. IL-8 further contributed to these associations, mediating roughly one-fourth of the effects of poor dental status on adverse OM-related outcomes. (**Figures 5D–L**).

## 4 Discussion

Our findings suggest that radiotherapy-induced OM may be better conceptualized not as an inevitable toxicity, but as a pathological process associated with the pre-existing oral ecological landscape. By employing trajectory modeling, clinical predictor analyses, and leveraging the natural experiment afforded by two ecologically distinct probiotics (SsM18 and SsK12), we observed that a dysbiotic, pro-inflammatory “ecological groundwork” was associated with a rapid-onset severe OM phenotype. Crucially, SsM18 use was associated with more favorable OM-related outcomes, consistent with its periodontal localization and ecological characteristics. Taken together, these findings are consistent with an ecological framework in which OM may involve interactions among host factors, inflammatory responses, and oral health status, rather than representing a purely epithelial injury. While the present study does not directly assess the oral microbiome or mechanistic pathways, our observations provide an exploratory clinical context interpretation and may help inform future hypothesis-driven studies focusing on cytokine signaling and immune modulation.

The trajectory modeling revealed two distinct clinical courses of radiotherapy-induced OM: one rapid-onset and severe, the other late-onset and mild. They diverged as early as the third week of radiotherapy (around the 15th fraction), which is a critical juncture where cumulative mucosal injury exceeds regenerative capacity. This early bifurcation was associated with baseline host and ecological characteristics present prior to radiotherapy initiation. Multivariate analysis identified poor dental status (BOAS–Teeth domain) and elevated IL-6 levels as independent predictors of the “rapid-onset, severe” trajectory. Together, these variables delineated a biologically fragile “groundwork” that predisposes the mucosa to accelerated breakdown once exposed to radiation stress.

Multivariate analysis identified two independent predictors of the “rapid-onset, severe” trajectory, each illuminating a component of the vulnerable ecological groundwork: namely, poor dental status, which represents dental plaque burden—a tangible indicator of microbial dysbiosis and acidic ecological imbalance within the oral cavity. It reflects a pre-existing microenvironment dominated by acidogenic and pro-inflammatory species, which diminishes epithelial resilience (57–60). The synergistic effect of this acid-producing environment with the radiotherapy-induced pH decrease further promotes the growth of acid-tolerant microbial communities and tissue irritation (61). Elevated cytokines capture a heightened systemic inflammatory tone. Radiotherapy-induced oxidative stress activates NF-κB and interferon-responsive pathways (62), resulting in the transcriptional upregulation of IL-6 and IL-8. Elevated IL-6 amplifies vascular permeability and matrix degradation (9, 63), whereas sustained IL-8 overexpression enhances neutrophil infiltration and ROS generation (64). This cytokine profile denotes a primed but dysregulated host immune state that amplifies neutrophil-driven oxidative stress upon radiation challenge. Another key, though non-independent, predictor was heavy alcohol consumption. As a known promoter of systemic oxidative stress, mucosal barrier dysfunction, salivary gland deterioration, oral environment degradation, and dysbiosis, it may further compounds the pro-inflammatory state (65–67).

Mediation analyses suggested that inflammatory biomarkers statistically mediated the observed associations between oral ecological factors and mucosal outcomes. Specifically, the association between poor dental status (BOAS–Teeth) and both OM severity and duration was partially mediated by elevated IL-6 levels, accounting for approximately 50–60% of the estimated association. In this framework, behavioral and ecological factors such as alcohol use and dental plaque may contribute to a chronic low-grade inflammatory milieu characterized by elevated IL-6 and IL-8, as supported by prior studies. Such an inflammatory background may be associated with reduced mucosal tolerance to radiotherapy-induced injury (3, 68, 69). In addition, mediation analyses indicated that probiotic supplementation was associated with shorter OM duration through reductions in IL-6 and IL-8, suggesting that microbial modulation may represent a relevant target for influencing inflammatory signaling pathways (70). These findings provide quantitative, hypothesis-generating evidence that a dysbiotic ecological groundwork may predispose mucosal injury through cytokine amplification and impaired epithelial repair, consistent with existing literature linking microbial cues to epithelial stress and immune regulation (9, 71). Together, these findings substantiate the concept that “the foundation determines the fate”—the pre-existing host–microbiome equilibrium dictates whether radiation exposure triggers controlled adaptation or rapid, destructive inflammation.

Given the central role of the periodontal niche in the pathogenesis of radiotherapy-induced OM, the superior efficacy of SsM18 may be related to its targeted modulation of this vulnerable ecological groundwork. In contrast to SsK12, which predominantly colonizes the pharyngeal and nasopharyngeal mucosa (72–77), SsM18 demonstrates a clear periodontal tropism, preferentially adhering to tooth enamel and gingival crevices (35). This localization allows SsM18 to act precisely at the “frontline” where plaque accumulation, acidic stress, and inflammatory activation converge (78). Mechanistically, unlike SsK12, which exhibits strong inhibitory activity against *Streptococcus pyogenes* (77, 79–82), SsM18 may exerts multi-dimensional ecological engineering. Through its bacteriocin repertoire (salivaricins A2, 9, MPS, and M) (36), it suppresses *Streptococcus mutans* and other acidogenic or pro-inflammatory taxa, thereby reducing plaque biomass and preventing dysbiotic biofilm maturation (83, 84). In parallel, its enzymatic activities, including dextranase and urease, can disrupt polysaccharide matrices and generate ammonia, contributing to local pH buffering and maintenance of near-neutral oral conditions (35, 78, 85, 86). These pH-modulating effects are physiologically significant, as lower oral pH (≤7.0) increases OM risk 2.3-fold, while pH ≤5.3 amplifies it 14–24-fold (87). Beyond these ecological effects, SsM18 may exhibit immunomodulatory capacity at the mucosal interface, attenuating NF-κB activation and suppressing pathogen-induced IL-6/IL-8 release in gingival fibroblasts (46, 49, 88). Taken together, these site-specific and immunological properties, supported by prior literature, are consistent with the hypothesis that SsM18 may contribute to stabilization of the oral microenvironment through modulation of plaque burden, local acidity, and inflammatory signaling (46, 49). This supports its characterization as a strain-specific, hypothesis-generating probiotic candidate rather than a nonspecific adjunct. In contrast, the predominantly oropharyngeal colonization of SsK12 may limit its influence on periodontal microecology (76, 77), a region increasingly recognized as central to mucositis pathogenesis. Together, these findings support a host–microbiome–inflammation framework in which pre-existing ecological vulnerability and inflammatory tone are associated with divergent mucosal responses to radiotherapy. Within this framework, targeted ecological modulation, as exemplified by SsM18, may represent a promising, hypothesis-generating strategy for the prevention of radiotherapy-induced OM.

## 5 Limitations

This study has several limitations. First, it was conducted prospectively with a pragmatic, non-randomized design, and the analyses were restricted to patients who adhered to the assigned recommendation, which may introduce selection bias and residual confounding. Moreover, outcome assessors were not formally blinded, and despite the use of standardized WHO and CTCAE criteria, assessment bias cannot be fully excluded. In this context, concomitant supportive therapies precluded completely isolating the independent probiotic effect. Taken together, these sources of bias limit causal inference and require cautious interpretation. Nevertheless, this pragmatic design reflects real-world clinical practice, where probiotic use is typically recommendation-based. Accordingly, the findings are exploratory and underscore the need for future randomized controlled trials with appropriate allocation concealment and intention-to-treat analyses. Second, as a single-center prospective cohort with a relatively modest sample size, a few unmeasured confounders cannot be fully excluded. For example, betel nut chewing and HPV status were not assessed due to regional clinical practice in northern China, and because only NPC patients were included, the generalizability of these findings to other head and neck cancer subtypes is limited and warrants site-specific validation. Third, Cytokines such as IL-6 and IL-8 are dynamically regulated, and single systemic measurements may not fully capture temporal changes or the local mucosal inflammatory milieu during radiotherapy. Accordingly, mediation findings should be interpreted as statistical rather than definitive biological mediation. Future studies incorporating longitudinal profiling of salivary cytokines are warranted to better characterize dynamic inflammatory pathways underlying OM. In addition, the relatively small sample size, limited outcome events, and fixed OM assessment schedule may have contributed to unstable estimates and wide 95% CIs, warranting cautious and exploratory interpretation. Finally, although potential mechanisms of SsM18 were discussed based on prior literature, oral microbiota composition and probiotic colonization were not directly assessed in this cohort. Accordingly, proposed ecological mechanisms should be interpreted as biologically plausible and hypothesis-generating rather than direct findings of the present study. As a purely clinical, exploratory investigation, future studies integrating oral microbiome metagenomics, salivary metabolomics, longitudinal salivary cytokine profiling, and experimental models are warranted to validate the microbial–immune pathways suggested by the current observations.

## 6 Conclusions and future directions

In conclusion, this study suggests that SsM18 supplementation may be associated with improved clinical outcomes of radiotherapy-induced OM in NPC. Changes in systemic inflammatory markers, including IL-6 and IL-8, were observed alongside clinical improvement. Although mechanistic pathways were not directly examined, the findings provide an exploratory clinical context for future studies investigating host–inflammation–microbiota interactions. Future randomized trials and mechanistic studies integrating cytokine profiling, microbiome sequencing, and mucosal transcriptomics are warranted to clarify whether changes in systemic cytokines translate into local mucosal immune regulation.

## Data Availability

The raw data supporting the conclusions of this article will be made available by the authors, without undue reservation.
