# Supplementary material for: Effectiveness of Streptococcus salivarius probiotics on alleviating radiation-induced oral mucositis via inflammatory and microecological modulation: a prospective pragmatic interventional study in nasopharyngeal carcinoma
Source: Front Immunol. 2026 Mar 3;17:1745549. doi: 10.3389/fimmu.2026.1745549 (PMC12992303; doi:10.3389/fimmu.2026.1745549)
Supplement: Supplementary file 1 [file Table1.docx]

S Table 1. Modified Beck Oral Assessment Scale (BOAS) for Oral Care Evaluation

| Area | Score | | | |
| --- | --- | --- | --- | --- |
|  | 1 | 2 | 3 | 4 |
| Lips | Smooth, pink, moist, and intact | Slightly dry, red | Dry, swollen isolated blisters | Edematous, inflamed blisters |
| Gingiva and oral mucosa | Smooth, pink, moist, and intact | Pale, dry, isolated lesions | Swollen red | Very dry and edematous, inflamed |
| Tongue | Smooth, pink, moist, and intact | Dry, prominent papillae | Dry, swollen, tip and papillae are red with lesions | Very dry, edematous, engorged coating |
| Teeth | Clean, no debris | Minimal debris | Moderate debris | Covered with debris |
| Saliva | Thin, watery plentiful | Increase in amount | Scanty and somewhat thicker | Thick and ropy, viscid or mucid |
| Total score | 5  No dysfunction  Minimum care every 12 h | 6-10  Mild dysfunction  Minimum care every 8-12 h | 11-15  Moderate dysfunction  Minimum care every 8 h | 16-20  Severe dysfunction  Minimum care every 4 h |

S Table 2. WHO Oral Mucositis (OM) Grading Criteria

| Grade | Symptoms |
| --- | --- |
| Grade 0 | No oral mucositis. |
| Grade 1 | Soreness ± erythema. |
| Grade 2 | Erythema, ulcers, solid diet possible. |
| Grade 3 | Ulcers, extensive erythema, liquid diet only. |
| Grade 4 | Alimentation not possible. |

S Table 3. Acute Radiation Toxicities Assessment (CTCAE v5.0)

| Domain | Grade | Detailed Criteria |
| --- | --- | --- |
| Dermatitis | 1 | Erythema or dry desquamation |
|  | 2 | Moderate to brisk erythema; patchy moist desquamation, mostly confined to skin folds and creases; moderate edema |
|  | 3 | Moist desquamation in areas other than skin folds and creases; bleeding induced by minor trauma or abrasion |
|  | 4 | Skin necrosis or ulceration of full-thickness dermis; life-threatening consequences |
|  | 5 | Death (rare) |
| Oral Mucositis | 1 | Asymptomatic or mild symptoms; intervention not indicated |
|  | 2 | Moderate pain or ulcer that need modified diet indicated (semi-liquid or soft food) |
|  | 3 | Severe pain; interfering with oral intake |
|  | 4 | Life-threatening consequences; urgent intervention indicated (Liquid food) |
|  | 5 | Death (rare) |
| Xerostomia | 1 | Symptomatic (e.g., dry or thick saliva) without significant dietary alteration; unstimulated saliva flow >0.2 ml/min |
|  | 2 | Symptomatic and significant oral intake alteration (e.g., copious water, other lubricants); unstimulated saliva 0.1-0.2 ml/min |
|  | 3 | Symptoms leading to inability to adequately aliment orally; IV fluids, tube feedings, or total parenteral nutrition indicated; unstimulated saliva <0.1 ml/min |
|  | 4 | Life-threatening complications, such as severe infection, mucosal ulcer, etc. |
|  | 5 | Death |
| Dysphagia | 1 | Symptomatic, able to eat regular diet |
|  | 2 | Symptomatic and altered eating/swallowing; modified diet indicated |
|  | 3 | Severely altered eating/swallowing; tube feeding, TPN, or hospitalization indicated |
|  | 4 | Life-threatening consequences; urgent intervention indicated |
|  | 5 | Death |
| Weight Loss | 1 | < 5% from baseline |
|  | 2 | 5-10% from baseline |
|  | 3 | 10-20% from baseline; nutritional support indicated |
|  | 4 | >20% from baseline; nutritional support indicated |
| Trismus | 1 | Jaw symptoms without impaired eating |
|  | 2 | Jaw symptoms with impaired eating |
|  | 3 | Jaw symptoms with inability to aliment orally |
| Neck soft tissue necrosis | 0 | None |
|  | 2 | Local wound care; medical intervention indicated (e.g., dressings or topical medications) |
|  | 3 | Operative debridement or other invasive intervention indicated (e.g., tissue reconstruction, flap, or grafting) |
|  | 4 | Life-threatening consequences; urgent intervention indicated |
|  | 5 | Death |

S Table 4. Adverse events accessed by CTCAE v5.0.

| Domain | Grade | Detailed Criteria |
| --- | --- | --- |
| Constipation (Gastrointestinal disorders) | 1 | Occasional or intermittent symptoms; occasional use of stool softeners, laxatives, dietary modification, or enema |
|  | 2 | Persistent symptoms with regular use of laxatives or enemas; limiting instrumental ADL |
|  | 3 | Obstipation with manual evacuation indicated; limiting selfcare ADL |
|  | 4 | Life-threatening consequences; urgent intervention indicated |
|  | 5 | Death (rare) |
| Diarrhea (Gastrointestinal disorders) | 1 | Increase of <4 stools per day over baseline; mild increase in ostomy output compared to baseline |
|  | 2 | Increase of 4 - 6 stools per day over baseline; moderate increase in ostomy output compared to baseline; limiting instrumental ADL |
|  | 3 | Increase of ≥7 stools per day over baseline; hospitalization indicated; severe increase in ostomy output compared to baseline; limiting selfcare ADL |
|  | 4 | Life-threatening consequences; urgent intervention indicated |
|  | 5 | Death (rare) |
| Gastritis (Gastrointestinal disorders) | 1 | Asymptomatic; clinical or diagnostic observations only; intervention not indicated |
|  | 2 | Symptomatic; altered GI function; medical intervention indicated |
|  | 3 | Severely altered eating or gastric function; TPN or hospitalization indicated |
|  | 4 | Life-threatening consequences; urgent operative intervention indicated |
|  | 5 | Death (rare) |
| Nausea (Gastrointestinal disorders) | 1 | Loss of appetite without alteration in eating habits |
|  | 2 | Oral intake decreased without significant weight loss, dehydration or malnutrition |
|  | 3 | Inadequate oral caloric or fluid intake; tube feeding, TPN, or hospitalization indicated |
| Vomiting (Gastrointestinal disorders) | 1 | Intervention not indicated |
|  | 2 | Outpatient IV hydration; medical intervention indicated |
|  | 3 | Tube feeding, TPN, or hospitalization indicated |
|  | 4 | Life-threatening consequences |
|  | 5 | Death (rare) |
| Anemia (Blood and lymphatic system disorders) | 1 | Hemoglobin (Hgb) <LLN - 10.0 g/dL; <LLN - 6.2 mmol/L; <LLN - 100 g/L |
|  | 2 | Hgb <10.0 - 8.0 g/dL; <6.2 - 4.9 mmol/L; <100 - 80g/L |
|  | 3 | Hgb <8.0 g/dL; <4.9 mmol/L; <80 g/L; transfusion indicated |
|  | 4 | Life-threatening consequences; urgent intervention indicated |
|  | 5 | Death (rare) |
| Febrile neutropenia (Blood and lymphatic system disorders) | 3 | ANC <1000/mm3 with a single temperature of >38.3 degrees C (101 degrees F) or a sustained temperature of >=38 degrees C (100.4 degrees F) for more than one hour |
|  | 4 | Life-threatening consequences; urgent intervention indicated |
|  | 5 | Death (rare) |
| Neutrophil count decreased (Blood and lymphatic system disorders) | 1 | <LLN - 1500/mm3; <LLN - 1.5 x 10e9 /L |
|  | 2 | <1500 - 1000/mm3; <1.5 - 1.0 x 10e9 /L |
|  | 3 | <1000 - 500/mm3; <1.0 - 0.5 x 10e9 /L |
|  | 4 | <500/mm3; <0.5 x 10e9 /L |
| Platelet count decreased (Blood and lymphatic system disorders) | 1 | <LLN - 75,000/mm3; <LLN - 75.0 x 10e9 /L |
|  | 2 | <75,000 - 50,000/mm3; <75.0 - 50.0 x 10e9 /L |
|  | 3 | <50,000 - 25,000/mm3; <50.0 - 25.0 x 10e9 /L |
|  | 4 | <25,000/mm3; <25.0 x 10e9 /L |
| White blood cell decreased (Blood and lymphatic system disorders) | 1 | <LLN - 3000/mm3; <LLN - 3.0 x 10e9 /L |
|  | 2 | <3000 - 2000/mm3; <3.0 - 2.0 x 10e9 /L |
|  | 3 | <2000 - 1000/mm3; <2.0 - 1.0 x 10e9 /L |
|  | 4 | <1000/mm3; <1.0 x 10e9 /L |

The overall severity of gastrointestinal toxicity and myelosuppression for each patient was determined by the highest-grade event recorded within each category. Gastrointestinal toxicity encompassed constipation, diarrhea, gastritis, nausea, and vomiting. Myelosuppression encompassed anemia, febrile neutropenia, and decreased counts of neutrophils, platelets, and white blood cells. ADL: Activities of Daily Living; GI: Gastrointestinal; TPN: Total Parenteral Nutrition; LLN: Lower Limit of Normal.

S Table 5. Late Radiation Toxicity Scoring Criteria (RTOG)

| Domain | Grade | Detailed Criteria |
| --- | --- | --- |
| Skin | 0 | None |
|  | 1 | Slight atrophy; pigmentation change; some hair loss |
|  | 2 | Patchy atrophy; moderate telangiectasia; total hair loss |
|  | 3 | Market atrophy; gross telangiectasia |
|  | 4 | Ulceration |
|  | 5 | Death |
| Neck Subcutaneous Tissue | 0 | No change over baseline |
|  | 1 | Slight fibrosis (induration) and slight loss of subcutaneous fat |
|  | 2 | Moderate fibrosis and atrophy; minimal field contracture |
|  | 3 | Severe induration and loss of subcutaneous tissue; field contracture |
|  | 4 | Necrosis |
|  | 5 | Death |
| Dysphagia | 0 | None |
|  | 1 | Able to swallow a regular diet but with occasional discomfort or mild difficulty. |
|  | 2 | Able to swallow soft foods but has difficulty with a regular diet; requires semi-liquid or soft foods. |
|  | 3 | Able to swallow liquid foods only. |
|  | 4 | Unable to aliment orally; requires nutritional support via nasogastric tube, gastrostomy, or intravenous nutrition. |
|  | 5 | Death |
| Hoarseness | 0 | None. |
|  | 1 | Mild hoarseness; able to speak normally and does not require therapeutic intervention. |
|  | 2 | Moderate hoarseness; affects speech clarity but still allows for daily conversation. |
|  | 3 | Severe hoarseness; difficulty speaking; require therapeutic intervention. |
|  | 4 | Profound hoarseness; or complete loss of voice, severely affected quality of life. |
|  | 5 | Death |
| Salivary glands | 0 | None |
|  | 1 | Slight dryness of mouth; good response on stimulation |
|  | 2 | Moderate dryness of mouth; poor response on stimulation |
|  | 3 | Severe telangiectasia Complete dryness of mouth; no Fibrosis response on stimulation |
|  | 4 | Fibrosis |
|  | 5 | Death |
| Trismus | 0 | None |
|  | 1 | Mild restriction of mouth opening; daily activities; inter-incisal opening >2 cm. |
|  | 2 | Moderate trismus; inter-incisal opening between 1.5 cm and 2 cm; begins to affect eating and speech |
|  | 3 | Severe trismus; inter-incisal opening ≤1.5 cm; severely impacts quality of life, requires medical intervention. |
|  | 4 | Profound trismus; nearly unable to open mouth, leading to severe difficulty eating and loss of speech function, may require surgery or other urgent therapeutic intervention. |
|  | 5 | Death. |
| Hearing Impairment | 0 | None |
|  | 1 | Mild hearing changes, such as reduced perception of high-frequency sounds, not significantly affecting daily communication. |
|  | 2 | Moderate hearing impairment; requiring increased attention especially in noisy settings; may require a hearing aid. |
|  | 3 | Severe hearing impairment; patient has obvious hearing difficulty even in quiet environments, daily communication is limited, requires reliance on hearing aids or other assistive devices. |
|  | 4 | Profound hearing impairment or deafness; complete loss of hearing, unable to communicate via normal means. |
|  | 5 | Death |
| Temporal Lobe Injury | 0 | None |
|  | 1 | Mild temporal lobe injury; may include minor memory problems or mild cognitive function changes, but does not affect daily activities. |
|  | 2 | Moderate temporal lobe injury; obvious memory impairment, cognitive decline, possibly accompanied by mild emotional or behavioral changes, affects daily life but does not require long-term medical intervention. |
|  | 3 | Severe temporal lobe injury; significant cognitive, emotional, or behavioral disturbances affecting daily living abilities; may require ongoing medical support or rehabilitation therapy. |
|  | 4 | Life-threatening temporal lobe injury; accompanied by seizures, severe behavioral abnormalities, or profound cognitive impairment, potentially leading to complete loss of independent living capacity, requires urgent intervention or long-term hospitalization. |
|  | 5 | Death |

S Table 6. Nutritional Risk Screening 2002 (NRS2002) Assessment Tool

| Impaired nutritional status | | Severity of disease (≈ stress metabolism) | |
| --- | --- | --- | --- |
| Absent Score 0 | Normal nutritional status | Absent Score 0 | Normal nutritional requirements |
| Mild Score 1 | Wt loss >5% in 3 months Or  Food intake below 50–75% of normal requirement in preceding week | Mild Score 1 | Hip fracture  Chronic patients, in particular with acute complications: cirrhosis,  COPD  Chronic hemodialysis, diabetes, oncology |
| Severe Score 3 | Wt loss >5% in 2 months Or  BMI 18.5–20.5+ impaired general condition O  Food intake 25–50% of normal requirement in preceding week | Severe Score 3 | Major abdominal surgery. Stroke  Severe pneumonia, hematologic malignancy |
| Severe Score 3 | Wt loss >5% in 1 month (≈ >15% in 3 months) Or  BMI <18.5+ impaired general condition Or  Food intake 0–25% of normal requirement in preceding week in preceding week | Severe Score 3 | Head injury  Bone marrow transplantation  Intensive care patients |
| Score: |  |  |  |
| Total score: |  |  |  |
| Calculate the total score:  1. Find score (0–3) for Impaired nutritional status (only one: choose the variable with highest score) and Severity of disease (≈ stress metabolism, i.e. increase in nutritional requirements).  2. Add the two scores (→ total score)  3. If age ≥70 years: add 1 to the total score to correct for frailty of elderly  4. If age-corrected total ≥3: start nutritional support | | | |

As a prototype, a patient with a score = 1 in severity of disease is admitted to hospital due to complications associated with a chronic disease. The patient is weak but out of bed regularly. Protein requirement is increased, but can be covered by oral diet or supplements in most cases. The prototype of score = 2 is a patient confined to bed due to illness, e.g. following major abdominal surgery or due to severe infection. Protein requirement is substantially increased but can be covered, although artificial feeding is required in many cases. The prototype of score = 3 is the intensive care patient with assisted ventilation, inotropic drugs, etc. Protein requirement is increased to the extent, that in most cases it cannot be covered by artificial feeding, but protein breakdown and N loss can be attenuated significantly.

S Table 7. Distribution of OM severity by group.

| Categories | Variables | | Total (n = 69) | | NC group (n = 23) | | K12 group (n = 23) | | M18 group (n = 23) | | *P* | | *P* (K12 vs NC) | | *P* (M18 vs NC) | | *P* (K12 vs M18) |
| --- | --- | --- | --- | --- | --- | --- | --- | --- | --- | --- | --- | --- | --- | --- | --- | --- | --- |
| WHO Grade (Wk1), n (%) | |  | |  | |  | |  | | 0.232* | | 1.000^§^ | | 0.232^§^ | | 0.232* | |
| 0 | | 63 (91.30) | | 20 (86.96) | | 20 (86.96) | | 23 (100.00) | |  | |  | |  | |  | |
| 1 | | 6 (8.70) | | 3 (13.04) | | 3 (13.04) | | 0 (0.00) | |  | |  | |  | |  | |
| WHO Grade (Wk2), n (%) | |  | |  | |  | |  | | 0.218* | | 0.905* | | 0.091* | | 0.195* | |
| 0 | | 39 (56.52) | | 10 (43.48) | | 12 (52.17) | | 17 (73.91) | |  | |  | |  | |  | |
| 1 | | 26 (37.68) | | 11 (47.83) | | 9 (39.13) | | 6 (26.09) | |  | |  | |  | |  | |
| 2 | | 4 (5.80) | | 2 (8.70) | | 2 (8.70) | | 0 (0.00) | |  | |  | |  | |  | |
| WHO Grade (Wk3), n (%) | |  | |  | |  | |  | | 0.078* | | 0.858* | | **0.022*** | | 0.085* | |
| 0 | | 9 (13.04) | | 2 (8.70) | | 2 (8.70) | | 5 (21.74) | |  | |  | |  | |  | |
| 1 | | 27 (39.13) | | 6 (26.09) | | 8 (34.78) | | 13 (56.52) | |  | |  | |  | |  | |
| 2 | | 26 (37.68) | | 10 (43.48) | | 11 (47.83) | | 5 (21.74) | |  | |  | |  | |  | |
| 3 | | 6 (8.70) | | 4 (17.39) | | 2 (8.70) | | 0 (0.00) | |  | |  | |  | |  | |
| 4 | | 1 (1.45) | | 1 (4.35) | | 0 (0.00) | | 0 (0.00) | |  | |  | |  | |  | |
| WHO Grade (Wk4), n (%) | |  | |  | |  | |  | | 0.269* | | 0.737* | | 0.244* | | 0.103* | |
| 0 | | 4 (5.80) | | 1 (4.35) | | 0 (0.00) | | 3 (13.04) | |  | |  | |  | |  | |
| 1 | | 15 (21.74) | | 3 (13.04) | | 5 (21.74) | | 7 (30.43) | |  | |  | |  | |  | |
| 2 | | 26 (37.68) | | 10 (43.48) | | 7 (30.43) | | 9 (39.13) | |  | |  | |  | |  | |
| 3 | | 18 (26.09) | | 6 (26.09) | | 8 (34.78) | | 4 (17.39) | |  | |  | |  | |  | |
| 4 | | 6 (8.70) | | 3 (13.04) | | 3 (13.04) | | 0 (0.00) | |  | |  | |  | |  | |
| WHO Grade (Wk5), n (%) | |  | |  | |  | |  | | 0.478* | | 0.572* | | 0.310* | | 0.465* | |
| 0 | | 3 (4.35) | | 1 (4.35) | | 0 (0.00) | | 2 (8.70) | |  | |  | |  | |  | |
| 1 | | 14 (20.29) | | 2 (8.70) | | 6 (26.09) | | 6 (26.09) | |  | |  | |  | |  | |
| 2 | | 27 (39.13) | | 9 (39.13) | | 8 (34.78) | | 10 (43.48) | |  | |  | |  | |  | |
| 3 | | 16 (23.19) | | 7 (30.43) | | 5 (21.74) | | 4 (17.39) | |  | |  | |  | |  | |
| 4 | | 9 (13.04) | | 4 (17.39) | | 4 (17.39) | | 1 (4.35) | |  | |  | |  | |  | |
| WHO Grade (Wk6), n (%) | |  | |  | |  | |  | | 0.534* | | 0.605* | | 0.327* | | 0.523* | |
| 0 | | 3 (4.35) | | 1 (4.35) | | 0 (0.00) | | 2 (8.70) | |  | |  | |  | |  | |
| 1 | | 18 (26.09) | | 3 (13.04) | | 7 (30.43) | | 8 (34.78) | |  | |  | |  | |  | |
| 2 | | 22 (31.88) | | 8 (34.78) | | 7 (30.43) | | 7 (30.43) | |  | |  | |  | |  | |
| 3 | | 17 (24.64) | | 7 (30.43) | | 5 (21.74) | | 5 (21.74) | |  | |  | |  | |  | |
| 4 | | 9 (13.04) | | 4 (17.39) | | 4 (17.39) | | 1 (4.35) | |  | |  | |  | |  | |
| WHO Grade (Wk7), n (%) | |  | |  | |  | |  | | 0.185* | | 0.802* | | 0.075* | | 0.239* | |
| 0 | | 13 (18.84) | | 2 (8.70) | | 3 (13.04) | | 8 (34.78) | |  | |  | |  | |  | |
| 1 | | 46 (66.67) | | 16 (69.57) | | 17 (73.91) | | 13 (56.52) | |  | |  | |  | |  | |
| 2 | | 10 (14.49) | | 5 (21.74) | | 3 (13.04) | | 2 (8.70) | |  | |  | |  | |  | |
| WHO Grade (Wk8), n (%) | |  | |  | |  | |  | | 0.679* | | 0.852* | | 0.487* | | 0.461* | |
| 0 | | 25 (36.23) | | 8 (34.78) | | 7 (30.43) | | 10 (43.48) | |  | |  | |  | |  | |
| 1 | | 36 (52.17) | | 11 (47.83) | | 13 (56.52) | | 12 (52.17) | |  | |  | |  | |  | |
| 2 | | 8 (11.59) | | 4 (17.39) | | 3 (13.04) | | 1 (4.35) | |  | |  | |  | |  | |
| WHO Grade (Wk33), n (%) | |  | |  | |  | |  | | 0.102^§^ | | 0.522^§^ | | **0.032**^§^ | | 0.243^§^ | |
| 0 | | 53 (76.81) | | 15 (65.22) | | 17 (73.91) | | 21 (91.30) | |  | |  | |  | |  | |
| 1 | | 16 (23.19) | | 8 (34.78) | | 6 (26.09) | | 2 (8.70) | |  | |  | |  | |  | |

S Table 8. Comparison of OM severity and other relevant metrics across study groups.

| Categories | Variables | Total (n = 69) | NC group (n = 23) | K12 group (n = 23) | M18 group (n = 23) | *P* | *P* (K12 vs NC) | *P* (M18 vs NC) | *P* (K12 vs M18) |
| --- | --- | --- | --- | --- | --- | --- | --- | --- | --- |
| WHO oral toxicity score | Occurrence of OM, n (%) |  |  |  |  | 0.768^*^ | 1.000^*^ | 1.000^*^ | 0.489^*^ |
|  | 0 | 3 (4.30) | 1 (4.30) | 0 (0.00) | 2 (8.70) |  |  |  |  |
|  | 1 | 22 (95.70) | 22 (95.70) | 23 (100.00) | 21 (91.30) |  |  |  |  |
|  |  |  |  |  |  |  |  |  |  |
|  | Occurrence of SOM, n (%) |  |  |  |  | 0.194^§^ | 0.555^§^ | 0.080^§^ | 0.227^§^ |
|  | 0 | 38 (55.07) | 10 (43.48) | 12 (52.17) | 16 (69.57) |  |  |  |  |
|  | 1 | 31 (44.93) | 13 (56.52) | 11 (47.83) | 7 (30.43) |  |  |  |  |
|  | Peak OM Grade | 2.00 (2.00, 3.00) | 3.00 (2.00,3.00) | 2.00 (2.00,3.00) | 2.00 (1.00,3.00) | 0.095^#^ | 0.599^+^ | **0.039**^+^ | 0.127^#^ |
|  | , M (Q₁, Q₃) |  |  |  |  |  |  |  |  |
|  | Radiotherapy Fractions to OM Onset | 11.00 (8.00, 14.00) | 10.00 (7.00,12.00) | 11.00 (8.50,13.00) | 14.00 (9.50,15.00) | **0.028**^#^ | 0.501^+^ | **0.014**^+^ | **0.046**^+^ |
|  | , M (Q₁, Q₃) |  |  |  |  |  |  |  |  |
|  | Total OM duration, M (Q₁, Q₃) | 71.00 (39.00, 108.00) | 93.00 (43.50,127.00) | 73.00 (38.50,113.50) | 47.00 (34.50,80.50) | 0.095^#^ | 0.575^+^ | **0.031**^+^ | 0.160^+^ |
|  | SOM duration, M (Q₁, Q₃) | 0.00 (0.00, 20.00) | 4.00 (0.00,24.50) | 0.00 (0.00,22.00) | 0.00 (0.00,4.50) | **0.046**^#^ | 0.744^+^ | **0.019**^+^ | **0.040**^+^ |
| CTCAE score at 15f | CTCAE score at 15f, M (Q₁, Q₃) | 5.00 (3.00, 6.00) | 6.00 (3.50,7.00) | 5.00 (4.00,6.00) | 4.00 (3.00,5.00) | **0.016**^#^ | 0.270^+^ | **0.009**^+^ | **0.038**^+^ |
|  | Oral mucositis (15f), n (%) |  |  |  |  | 0.098* | 0.771* | **0.022*** | 0.150* |
|  | 0 | 9 (13.04) | 2 (8.70) | 2 (8.70) | 5 (21.74) |  |  |  |  |
|  | 1 | 28 (40.58) | 6 (26.09) | 9 (39.13) | 13 (56.52) |  |  |  |  |
|  | 2 | 25 (36.23) | 10 (43.48) | 10 (43.48) | 5 (21.74) |  |  |  |  |
|  | 3 | 6 (8.70) | 4 (17.39) | 2 (8.70) | 0 (0.00) |  |  |  |  |
|  | 4 | 1 (1.45) | 1 (4.35) | 0 (0.00) | 0 (0.00) |  |  |  |  |
|  | Dysphagia (15f), n (%) |  |  |  |  | 0.058* | 0.812* | **0.024*** | 0.102* |
|  | 0 | 9 (13.04) | 2 (8.70) | 2 (8.70) | 5 (21.74) |  |  |  |  |
|  | 1 | 28 (40.58) | 6 (26.09) | 9 (39.13) | 13 (56.52) |  |  |  |  |
|  | 2 | 31 (44.93) | 14 (60.87) | 12 (52.17) | 5 (21.74) |  |  |  |  |
|  | 3 | 1 (1.45) | 1 (4.35) | 0 (0.00) | 0 (0.00) |  |  |  |  |
|  | Weight loss (15f), n (%) |  |  |  |  | 0.656* | 1.000^§^ | 0.345^§^ | 0.530* |
|  | 0 | 45 (65.22) | 14 (60.87) | 14 (60.87) | 17 (73.91) |  |  |  |  |
|  | 1 | 23 (33.33) | 9 (39.13) | 8 (34.78) | 6 (26.09) |  |  |  |  |
|  | 2 | 1 (1.45) | 0 (0.00) | 1 (4.35) | 0 (0.00) |  |  |  |  |
|  | Radiotherapy -induced xerostomia (15f), n (%) |  |  |  |  | 0.775* | 1.000* | 0.435* | 0.774* |
|  | 0 | 9 (13.04) | 3 (13.04) | 3 (13.04) | 3 (13.04) |  |  |  |  |
|  | 1 | 30 (43.48) | 10 (43.48) | 10 (43.48) | 10 (43.48) |  |  |  |  |
|  | 2 | 26 (37.68) | 10 (43.48) | 9 (39.13) | 7 (30.43) |  |  |  |  |
|  | 3 | 4 (5.80) | 0 (0.00) | 1 (4.35) | 3 (13.04) |  |  |  |  |
|  | Radiotherapy -induced trismus (15f), n (%) |  |  |  |  | 0.313* | 0.608* | 0.233* | 1.000* |
|  | 0 | 65 (94.20) | 20 (86.96) | 22 (95.65) | 23 (100.00) |  |  |  |  |
|  | 1 | 3 (4.35) | 2 (8.70) | 1 (4.35) | 0 (0.00) |  |  |  |  |
|  | 2 | 1 (1.45) | 1 (4.35) | 0 (0.00) | 0 (0.00) |  |  |  |  |
| CTCAE score at 33f | CTCAE score at 33f, M (Q₁, Q₃) | 9.00 (6.00, 11.00) | 10.00 (7.00,11.00) | 9.00 (6.50,11.00) | 9.00 (5.00,10.50) | 0.545^#^ | 0.799^+^ | 0.310^+^ | 0.414^+^ |
|  | Oral mucositis (33f), n (%) |  |  |  |  | 0.680* | 0.790* | 0.456* | 0.523* |
|  | 0 | 3 (4.35) | 1 (4.35) | 0 (0.00) | 2 (8.70) |  |  |  |  |
|  | 1 | 19 (27.54) | 4 (17.39) | 7 (30.43) | 8 (34.78) |  |  |  |  |
|  | 2 | 21 (30.43) | 7 (30.43) | 7 (30.43) | 7 (30.43) |  |  |  |  |
|  | 3 | 17 (24.64) | 7 (30.43) | 5 (21.74) | 5 (21.74) |  |  |  |  |
|  | 4 | 9 (13.04) | 4 (17.39) | 4 (17.39) | 1 (4.35) |  |  |  |  |
|  | Neck soft tissue necrosis (33f), n (%) |  |  |  |  | 1.000* | 1.000^§^ | 1.000* | 1.000^§^ |
|  | 0 | 62 (89.86) | 21 (91.30) | 20 (86.96) | 21 (91.30) |  |  |  |  |
|  | 1 | 7 (10.14) | 2 (8.70) | 3 (13.04) | 2 (8.70) |  |  |  |  |
|  | Dysphagia (33f), n (%) |  |  |  |  | 0.454* | 0.656* | 0.321* | 0.360* |
|  | 0 | 3 (4.35) | 1 (4.35) | 0 (0.00) | 2 (8.70) |  |  |  |  |
|  | 1 | 19 (27.54) | 4 (17.39) | 7 (30.43) | 8 (34.78) |  |  |  |  |
|  | 2 | 38 (55.07) | 14 (60.87) | 12 (52.17) | 12 (52.17) |  |  |  |  |
|  | 3 | 9 (13.04) | 4 (17.39) | 4 (17.39) | 1 (4.35) |  |  |  |  |
|  | Dermatitis (33f), n (%) |  |  |  |  | 0.992* | 0.860* | 1.000* | 1.000* |
|  | 0 | 18 (26.09) | 7 (30.43) | 5 (21.74) | 6 (26.09) |  |  |  |  |
|  | 1 | 33 (47.83) | 11 (47.83) | 11 (47.83) | 11 (47.83) |  |  |  |  |
|  | 2 | 16 (23.19) | 5 (21.74) | 6 (26.09) | 5 (21.74) |  |  |  |  |
|  | 3 | 2 (2.90) | 0 (0.00) | 1 (4.35) | 1 (4.35) |  |  |  |  |
|  | Weight loss (33f), n (%) |  |  |  |  | 0.885* | 0.667* | 0.782* | 0.894* |
|  | 0 | 6 (8.70) | 1 (4.35) | 2 (8.70) | 3 (13.04) |  |  |  |  |
|  | 1 | 17 (24.64) | 5 (21.74) | 7 (30.43) | 5 (21.74) |  |  |  |  |
|  | 2 | 33 (47.83) | 11 (47.83) | 11 (47.83) | 11 (47.83) |  |  |  |  |
|  | 3 | 13 (18.84) | 6 (26.09) | 3 (13.04) | 4 (17.39) |  |  |  |  |
|  | Radiotherapy -induced xerostomia (33f), n (%) |  |  |  |  | 0.346^§^ | 0.192^§^ | 0.798^§^ | 0.222^§^ |
|  | 1 | 21 (30.43) | 8 (34.78) | 4 (17.39) | 9 (39.13) |  |  |  |  |
|  | 2 | 31 (44.93) | 8 (34.78) | 14 (60.87) | 9 (39.13) |  |  |  |  |
|  | 3 | 17 (24.64) | 7 (30.43) | 5 (21.74) | 5 (21.74) |  |  |  |  |
|  | Radiotherapy -induced trismus (33f), n (%) |  |  |  |  | 0.482* | 1.000^§^ | 0.412^§^ | 0.412* |
|  | 0 | 60 (86.96) | 21 (91.30) | 21 (91.30) | 18 (78.26) |  |  |  |  |
|  | 1 | 9 (13.04) | 2 (8.70) | 2 (8.70) | 5 (21.74) |  |  |  |  |
| After Radiotherapy | RTOG score, M (Q₁, Q₃) | 4.00 (3.00, 6.00) | 5.00 (3.00,7.50) | 4.00 (3.00,5.50) | 4.00 (2.50,5.00) | 0.304^#^ | 0.232^+^ | 0.162^+^ | 0.747^+^ |
|  | Skin reaction (after radiotherapy), n (%) |  |  |  |  | 0.989* | 1.000* | 0.917* | 0.950* |
|  | 0 | 7 (10.14) | 3 (13.04) | 2 (8.70) | 2 (8.70) |  |  |  |  |
|  | 1 | 33 (47.83) | 11 (47.83) | 12 (52.17) | 10 (43.48) |  |  |  |  |
|  | 2 | 26 (37.68) | 8 (34.78) | 8 (34.78) | 10 (43.48) |  |  |  |  |
|  | 3 | 3 (4.35) | 1 (4.35) | 1 (4.35) | 1 (4.35) |  |  |  |  |
|  | Neck subcutaneous tissue (after radiotherapy), n (%) |  |  |  |  | 1.000* | 1.000* | 1.000^§^ | 1.000^§^ |
|  | 0 | 62 (89.86) | 21 (91.30) | 20 (86.96) | 21 (91.30) |  |  |  |  |
|  | 1 | 7 (10.14) | 2 (8.70) | 3 (13.04) | 2 (8.70) |  |  |  |  |
|  | Dysphagia (after radiotherapy), n (%) |  |  |  |  | 0.255^§^ | 0.340* | 0.131^§^ | 0.510* |
|  | 0 | 16 (23.19) | 5 (21.74) | 4 (17.39) | 7 (30.43) |  |  |  |  |
|  | 1 | 36 (52.17) | 9 (39.13) | 14 (60.87) | 13 (56.52) |  |  |  |  |
|  | 2 | 17 (24.64) | 9 (39.13) | 5 (21.74) | 3 (13.04) |  |  |  |  |
|  | Radiotherapy -induced hoarseness (after radiotherapy), n (%) |  |  |  |  | 0.294* | 0.373* | 0.094* | 0.915* |
|  | 0 | 30 (43.48) | 6 (26.09) | 11 (47.83) | 13 (56.52) |  |  |  |  |
|  | 1 | 23 (33.33) | 8 (34.78) | 8 (34.78) | 7 (30.43) |  |  |  |  |
|  | 2 | 12 (17.39) | 6 (26.09) | 3 (13.04) | 3 (13.04) |  |  |  |  |
|  | 3 | 4 (5.80) | 3 (13.04) | 1 (4.35) | 0 (0.00) |  |  |  |  |
|  | Salivary glands (after radiotherapy), n (%) |  |  |  |  | 0.415* | 0.695* | 0.170* | 0.581* |
|  | 0 | 10 (14.49) | 3 (13.04) | 4 (17.39) | 3 (13.04) |  |  |  |  |
|  | 1 | 45 (65.22) | 13 (56.52) | 14 (60.87) | 18 (78.26) |  |  |  |  |
|  | 2 | 13 (18.84) | 7 (30.43) | 4 (17.39) | 2 (8.70) |  |  |  |  |
|  | 3 | 1 (1.45) | 0 (0.00) | 1 (4.35) | 0 (0.00) |  |  |  |  |
|  | Radiotherapy -induced trismus (after radiotherapy), n (%) |  |  |  |  | 0.566^§^ | 1.000^§^ | 0.502^§^ | 0.300^§^ |
|  | 0 | 53 (76.81) | 18 (78.26) | 19 (82.61) | 16 (69.57) |  |  |  |  |
|  | 1 | 16 (23.19) | 5 (21.74) | 4 (17.39) | 7 (30.43) |  |  |  |  |
|  | Radiotherapy -induced hearing impairment (after radiotherapy), n (%) |  |  |  |  | 1.000* | 1.000^§^ | 1.000^§^ | 1.000^§^ |
|  | 0 | 61 (88.41) | 20 (86.96) | 21 (91.30) | 20 (86.96) |  |  |  |  |
|  | 1 | 8 (11.59) | 3 (13.04) | 2 (8.70) | 3 (13.04) |  |  |  |  |
| Xerostomia | Radiotherapy -induced xerostomia (Wk1), n (%) |  |  |  |  | 0.508^§^ | 0.326* | 1.000^§^ | 0.326^§^ |
|  | 0 | 51 (73.91) | 18 (78.26) | 15 (65.22) | 18 (78.26) |  |  |  |  |
|  | 1 | 18 (26.09) | 5 (21.74) | 8 (34.78) | 5 (21.74) |  |  |  |  |
|  | Radiotherapy -induced xerostomia (Wk2), n (%) |  |  |  |  | 0.659* | 0.325* | 0.615* | 1.000* |
|  | 0 | 26 (37.68) | 11 (47.83) | 7 (30.43) | 8 (34.78) |  |  |  |  |
|  | 1 | 33 (47.83) | 8 (34.78) | 13 (56.52) | 12 (52.17) |  |  |  |  |
|  | 2 | 10 (14.49) | 4 (17.39) | 3 (13.04) | 3 (13.04) |  |  |  |  |
|  | Radiotherapy -induced xerostomia (Wk3), n (%) |  |  |  |  | 0.744* | 0.692* | 0.435* | 1.000* |
|  | 0 | 8 (11.59) | 3 (13.04) | 2 (8.70) | 3 (13.04) |  |  |  |  |
|  | 1 | 31 (44.93) | 10 (43.48) | 11 (47.83) | 10 (43.48) |  |  |  |  |
|  | 2 | 25 (36.23) | 10 (43.48) | 8 (34.78) | 7 (30.43) |  |  |  |  |
|  | 3 | 5 (7.25) | 0 (0.00) | 2 (8.70) | 3 (13.04) |  |  |  |  |
|  | Radiotherapy -induced xerostomia (Wk4), n (%) |  |  |  |  | 0.728* | 0.840* | 0.793* | 0.376* |
|  | 0 | 1 (1.45) | 0 (0.00) | 0 (0.00) | 1 (4.35) |  |  |  |  |
|  | 1 | 25 (36.23) | 9 (39.13) | 8 (34.78) | 8 (34.78) |  |  |  |  |
|  | 2 | 33 (47.83) | 11 (47.83) | 13 (56.52) | 9 (39.13) |  |  |  |  |
|  | 3 | 10 (14.49) | 3 (13.04) | 2 (8.70) | 5 (21.74) |  |  |  |  |
|  | Radiotherapy -induced xerostomia (Wk5), n (%) |  |  |  |  | 0.390^§^ | 0.285^§^ | 0.943^§^ | 0.161^§^ |
|  | 1 | 21 (30.43) | 8 (34.78) | 4 (17.39) | 9 (39.13) |  |  |  |  |
|  | 2 | 31 (44.93) | 9 (39.13) | 14 (60.87) | 8 (34.78) |  |  |  |  |
|  | 3 | 17 (24.64) | 6 (26.09) | 5 (21.74) | 6 (26.09) |  |  |  |  |
|  | Radiotherapy -induced xerostomia (Wk6), n (%) |  |  |  |  | 0.158* | 0.123^§^ | 0.757^§^ | 0.071* |
|  | 0 | 1 (1.45) | 0 (0.00) | 1 (4.35) | 0 (0.00) |  |  |  |  |
|  | 1 | 21 (30.43) | 8 (34.78) | 3 (13.04) | 10 (43.48) |  |  |  |  |
|  | 2 | 30 (43.48) | 8 (34.78) | 14 (60.87) | 8 (34.78) |  |  |  |  |
|  | 3 | 17 (24.64) | 7 (30.43) | 5 (21.74) | 5 (21.74) |  |  |  |  |
|  | Radiotherapy -induced xerostomia (Wk7), n (%) |  |  |  |  | 0.720* | 0.904* | 0.443* | 0.283* |
|  | 0 | 4 (5.80) | 1 (4.35) | 1 (4.35) | 2 (8.70) |  |  |  |  |
|  | 1 | 38 (55.07) | 11 (47.83) | 12 (52.17) | 15 (65.22) |  |  |  |  |
|  | 2 | 24 (34.78) | 10 (43.48) | 8 (34.78) | 6 (26.09) |  |  |  |  |
|  | 3 | 3 (4.35) | 1 (4.35) | 2 (8.70) | 0 (0.00) |  |  |  |  |
|  | Radiotherapy -induced xerostomia (Wk8), n (%) |  |  |  |  | 0.282* | 0.472^§^ | 0.099^§^ | 0.504* |
|  | 0 | 14 (20.29) | 4 (17.39) | 4 (17.39) | 6 (26.09) |  |  |  |  |
|  | 1 | 41 (59.42) | 11 (47.83) | 15 (65.22) | 15 (65.22) |  |  |  |  |
|  | 2 | 14 (20.29) | 8 (34.78) | 4 (17.39) | 2 (8.70) |  |  |  |  |
|  | Radiotherapy -induced xerostomia (Wk33), n (%) |  |  |  |  | 0.406^§^ | 0.522^§^ | 0.179^§^ | 0.623* |
|  | 0 | 51 (73.91) | 15 (65.22) | 17 (73.91) | 19 (82.61) |  |  |  |  |
|  | 1 | 18 (26.09) | 8 (34.78) | 6 (26.09) | 4 (17.39) |  |  |  |  |
| Relevant Clinical Indicators | CYFRA21-1 (after radiotherapy), M (Q₁, Q₃) | 1.67 (1.33, 2.47) | 1.58 (1.23,2.59) | 1.52 (1.33,2.04) | 2.08 (1.39,2.56) | 0.288^#^ | 0.546^+^ | 0.410^+^ | 0.106^+^ |
|  | CEA (after radiotherapy), M (Q₁, Q₃) | 1.24 (0.81, 2.03) | 1.24 (0.70,1.96) | 1.18 (0.82,1.74) | 1.64 (0.96,2.58) | 0.260^#^ | 0.218^+^ | 0.262^+^ | 0.095^+^ |
|  | SCC (after radiotherapy), M (Q₁, Q₃) | 1.66 (1.35, 2.49) | 1.47 (1.34,2.48) | 1.63 (1.29,2.48) | 1.83 (1.53,2.42) | 0.637^#^ | 0.921^+^ | 0.503^+^ | 0.356^+^ |
|  | IL6, M (Q₁, Q₃) | 2.57 (1.73, 5.97) | 3.58 (2.13,12.51) | 2.53 (1.81,5.95) | 1.89 (1.50,3.67) | 0.057^#^ | 0.213^+^ | **0.020**^+^ | 0.214^+^ |
|  | IL8, M (Q₁, Q₃) | 0.82 (0.58, 2.75) | 1.24 (0.66,3.97) | 1.38 (0.53,4.08) | 0.71 (0.59,1.25) | 0.286^#^ | 0.489^+^ | 0.111^+^ | 0.423^+^ |
|  | IFNα, M (Q₁, Q₃) | 1.49 (0.88, 1.73) | 1.59 (1.29,1.77) | 1.45 (1.21,1.72) | 1.34 (0.77,1.72) | 0.266^#^ | 0.435^+^ | 0.119^+^ | 0.362^+^ |
|  | IFNγ, M (Q₁, Q₃) | 2.01 (1.69, 2.56) | 2.15 (1.72,2.73) | 1.84 (1.69,2.96) | 1.92 (1.64,2.25) | 0.566^#^ | 0.758^+^ | 0.221^+^ | 0.709^+^ |
|  | TNF, M (Q₁, Q₃) | 1.78 (1.42, 2.48) | 2.63 (1.86,3.58) | 1.73 (1.15,1.90) | 1.78 (1.49,2.13) | **<.001**^#^ | **<.001**^+^ | **0.005**^+^ | 0.239^+^ |
|  | Neutrophil count (after radiotherapy), M (Q₁, Q₃) | 3.11 (2.38, 3.91) | 2.74 (2.41,3.42) | 2.96 (2.08,3.77) | 3.75 (2.61,4.43) | 0.126^#^ | 0.809^+^ | 0.091^+^ | 0.073^+^ |
|  | Lymphocyte count (after radiotherapy), M (Q₁, Q₃) | 0.72 (0.54, 0.84) | 0.73 (0.56,0.83) | 0.75 (0.52,0.89) | 0.70 (0.54,0.82) | 0.887^#^ | 1.000^+^ | 0.700^+^ | 0.660^+^ |
|  | Monocyte count (after radiotherapy), M (Q₁, Q₃) | 0.42 (0.35, 0.51) | 0.37 (0.30,0.56) | 0.42 (0.37,0.49) | 0.42 (0.40,0.58) | 0.416^#^ | 0.792^+^ | 0.223^+^ | 0.317^+^ |
|  | Eosinophil count (after radiotherapy), M (Q₁, Q₃) | 0.07 (0.04, 0.13) | 0.06 (0.04,0.11) | 0.07 (0.04,0.10) | 0.10 (0.06,0.15) | 0.097^#^ | 0.956^+^ | 0.094^+^ | **0.042**^+^ |
|  | Basophil count (after radiotherapy), M (Q₁, Q₃) | 0.02 (0.01, 0.03) | 0.02 (0.01,0.03) | 0.01 (0.01,0.02) | 0.02 (0.01,0.04) | 0.068^#^ | **0.041**^+^ | 0.812^+^ | 0.052^+^ |
|  | Hb count (after radiotherapy), M (Q₁, Q₃) | 132.00 (121.00, 140.00) | 133.00 (123.50,144.00) | 128.00 (119.00,137.00) | 132.00 (120.50,142.00) | 0.390^#^ | 0.180^+^ | 0.692^+^ | 0.373^+^ |
|  | Platelet count (after radiotherapy), M (Q₁, Q₃) | 206.00 (172.00, 243.00) | 237.00 (191.00,283.50) | 183.00 (156.00,212.50) | 206.00 (174.50,233.50) | 0.053^#^ | **0.019**^+^ | 0.503^+^ | 0.102^+^ |
|  | Albumin (after radiotherapy), M (Q₁, Q₃) | 41.80 (39.60, 44.10) | 40.90 (39.60,43.85) | 41.40 (40.20,44.50) | 42.60 (39.35,44.95) | 0.780^#^ | 0.468^+^ | 0.644^+^ | 0.991^+^ |
|  | NLR (after radiotherapy), M (Q₁, Q₃) | 4.46 (3.23, 6.14) | 4.34 (3.09,6.30) | 4.28 (2.97,4.99) | 5.18 (3.50,6.72) | 0.380^#^ | 0.648^+^ | 0.423^+^ | 0.161^+^ |
|  | PLR (after radiotherapy), M (Q₁, Q₃) | 310.00 (229.75, 437.14) | 329.11 (261.86,436.38) | 254.93 (177.06,376.81) | 333.33 (237.14,511.17) | 0.180^#^ | 0.083^+^ | 0.974^+^ | 0.155^+^ |
|  | LMR (after radiotherapy), M (Q₁, Q₃) | 1.72 (1.10, 2.35) | 1.89 (1.12,2.68) | 1.92 (1.27,2.44) | 1.65 (1.08,1.82) | 0.366^#^ | 0.991^+^ | 0.339^+^ | 0.147^+^ |
|  | SII (after radiotherapy), M (Q₁, Q₃) | 875.59 (596.25, 1329.28) | 922.83 (684.74,1382.49) | 756.93 (522.59,1002.43) | 952.47 (645.72,2100.67) | 0.147^#^ | 0.168^+^ | 0.531^+^ | 0.068^+^ |
|  | SIRI (after radiotherapy), M (Q₁, Q₃) | 1.88 (1.31, 2.86) | 1.80 (1.04,2.60) | 1.85 (1.31,2.13) | 2.20 (1.50,3.90) | 0.209^#^ | 0.744^+^ | 0.223^+^ | 0.083^+^ |
|  | PNI (after radiotherapy), M (Q₁, Q₃) | 45.05 (43.15, 48.25) | 44.55 (43.12,47.98) | 46.40 (43.90,48.00) | 44.75 (42.73,49.03) | 0.793^#^ | 0.546^+^ | **0.921**^+^ | 0.590^+^ |
|  | Δ albumin, M (Q₁, Q₃) | 1.60 (0.00, 3.00) | 2.10 (0.45,3.35) | 1.20 (-0.70,2.45) | 1.70 (0.80,3.10) | 0.436^#^ | 0.227^+^ | 0.800^+^ | 0.345^+^ |
| Other | Hormone use days during radiotherapy, M (Q₁, Q₃) | 0.00 (0.00, 4.00) | 1.00 (0.00,9.00) | 0.00 (0.00,4.00) | 0.00 (0.00,0.00) | **0.037**^#^ | 0.205^+^ | **0.010**^+^ | 0.228^+^ |
|  | Nutritional support during radiotherapy | 0.00 (0.00, 0.00) | 0.00 (0.00,0.00) | 0.00 (0.00,0.00) | 0.00 (0.00,0.00) | 0.397^#^ | 0.988^+^ | 0.232^+^ | 0.212^+^ |
|  | , M (Q₁, Q₃) |  |  |  |  |  |  |  |  |
|  | Total weight lost, M (Q₁, Q₃) | 4.50 (3.00, 6.00) | 4.00 (2.50,6.00) | 4.50 (3.00,5.25) | 4.50 (3.00,7.00) | 0.917^#^ | 0.783^+^ | 0.700^+^ | 0.895 |
|  | Hypogeusia, n (%) |  |  |  |  | 0.907* | 1.000* | 0.910* | 0.695* |
|  | 3 | 10 (14.49) | 3 (13.04) | 4 (17.39) | 3 (13.04) |  |  |  |  |
|  | 4 | 16 (23.19) | 5 (21.74) | 4 (17.39) | 7 (30.43) |  |  |  |  |
|  | 5 | 43 (62.32) | 15 (65.22) | 15 (65.22) | 13 (56.52) |  |  |  |  |
|  | Radiotherapy interruption, n (%) |  |  |  |  | 0.768* | 1.000^§^ | 0.470^§^ | 1.000* |
|  | 0 | 66 (95.65) | 21 (91.30) | 22 (95.65) | 23 (100.00) |  |  |  |  |
|  | 1 | 3 (4.35) | 2 (8.70) | 1 (4.35) | 0 (0.00) |  |  |  |  |
|  | Myelosuppression, n (%) |  |  |  |  | 0.493* | 0.863* | 0.190* | 0.535* |
|  | 0 | 43 (62.32) | 12 (52.17) | 14 (60.87) | 17 (73.91) |  |  |  |  |
|  | 1 | 18 (26.09) | 7 (30.43) | 7 (30.43) | 4 (17.39) |  |  |  |  |
|  | 2 | 4 (5.80) | 3 (13.04) | 1 (4.35) | 0 (0.00) |  |  |  |  |
|  | 3 | 4 (5.80) | 1 (4.35) | 1 (4.35) | 2 (8.70) |  |  |  |  |
|  | Hepatic Toxicity, n (%) |  |  |  |  | 1.000* | 1.000* | 1.000^§^ | 1.000* |
|  | 0 | 67 (97.10) | 22 (95.65) | 23 (100.00) | 22 (95.65) |  |  |  |  |
|  | 1 | 2 (2.90) | 1 (4.35) | 0 (0.00) | 1 (4.35) |  |  |  |  |
|  | Gastrointestinal reaction (15f), n (%) |  |  |  |  | **0.046*** | 0.200* | **0.030*** | 0.674* |
|  | 0 | 49 (71.01) | 12 (52.17) | 17 (73.91) | 20 (86.96) |  |  |  |  |
|  | 1 | 17 (24.64) | 8 (34.78) | 6 (26.09) | 3 (13.04) |  |  |  |  |
|  | 2 | 3 (4.35) | 3 (13.04) | 0 (0.00) | 0 (0.00) |  |  |  |  |
|  | Painkiller Use, n (%) |  |  |  |  | 0.406^§^ | 0.522^§^ | 0.179^§^ | 0.475^§^ |
|  | 0 | 51 (73.91) | 15 (65.22) | 17 (73.91) | 19 (82.61) |  |  |  |  |
|  | 1 | 18 (26.09) | 8 (34.78) | 6 (26.09) | 4 (17.39) |  |  |  |  |

#: Kruskal-waills test, §: Chi-square test, *: Fisher exact, +: Mann-Whitney test

M: Median, Q₁: 1st Quartile, Q₃: 3st Quartile; WHO Grade: WHO oral toxicity score; Wk: weekend; CTCAE: Common Terminology Criteria for Adverse Events; 15f: At fraction 15; 33f: At fraction 33; RTOG: Radiation Therapy Oncology Group Score; SFR: Salivary flow rate; CYFRA21-1: Cytokeratin 19 fragment antigen 21-1; CEA: Carcinoembryonic antigen; SCC: Squamous cell carcinoma antigen; IL: Interleukin; IFN: Interferon; TNF: Tumor Necrosis Factor; NLR: Neutrophil-to-lymphocyte ratio; PLR: Platelet-to-lymphocyte ratio; LMR: Lymphocyte-to-monocyte ratio; SII: Systemic inflammatory index; SIRI: Systemic inflammatory response index; PNI: Prognostic nutritional index

S Table 9. Fit indices for group-based trajectory modeling of WHO Oral Toxicity score.

| Number of  trajectory  groups | Polynomial function order | BIC | APPA | Cubic | | OCC | | Proportions in each trajectory group (%) | | |
| --- | --- | --- | --- | --- | --- | --- | --- | --- | --- | --- |
|  |  |  | mean | Estimate | *P* | Mean | The lowest value  across group(s) | 1 | 2 | 3 |
| 1 | Score of WHO Oral Toxicity score (3) | -763.97 | - | 0.008 | <0.001 | NA | NA | 100% | - | - |
| 2 | Score of WHO Oral Toxicity score (3 3) | -648.21 | 0.92 | 0.008/0.0081 | <0.001/<0.001 | 43.30 | 38.35 | 53.6% | 46.3% | - |
| 3 | Score of WHO Oral Toxicity score (3 3 3) | -658.80 | 0.33 | 0.008/0.0081/0.0195 | <0.001/<0.001/1.000 | 48.25 | 38.35 | 53.6% | 46.3% | 0 |

The model that best fit our data is shown in bold. 1 Polynomial function order: 1 = linear, 2 = quadratic, 3 = cubic.

APPA=average posterior probability of assignment. BIC=Bayesian information criterion. IC=intrinsic capacity. NA=not applicable. OCC=odds of correct classification.

S Table 10. Univariate analysis of factors associated with OM trajectories.

| Variables | Level | β | SE | Z | *P* | OR (95%CI) |
| --- | --- | --- | --- | --- | --- | --- |
| Age |  | 0.011 | 0.016 | 0.692 | 0.4891 | 1.01 (0.98 - 1.04) |
| Albumin |  | -0.196 | 0.093 | -2.113 | 0.0346 | 0.82 (0.68 - 0.98) |
| Weekly standard drinks |  | 0.098 | 0.034 | 2.869 | **0.004** | 1.10 (1.04 - 1.19) |
| Basophil |  | -0.693 | 3.354 | -0.207 | 0.8364 | 0.50 (<0.001 - 474.54) |
| BOAS |  | 1.856 | 0.429 | 4.323 | **<0.001** | 6.40 (3.03 - 16.63) |
| BMI |  | -0.059 | 0.056 | -1.061 | 0.2887 | 0.94 (0.84 - 1.05) |
| CEA |  | -0.09 | 0.125 | -0.718 | 0.4725 | 0.91 (0.69 - 1.15) |
| Clinical Stage | 3 |  |  |  |  | 1.00 (Reference) |
|  | 4 | -0.089 | 0.486 | -0.183 | 0.8549 | 0.92 (0.35 - 2.37) |
| CYF |  | -0.006 | 0.043 | -0.135 | 0.8924 | 0.99 (0.91 - 1.08) |
| Δ Albumin |  | 0.111 | 0.081 | 1.359 | 0.1741 | 1.12 (0.96 - 1.33) |
| Diabetes | 0 |  |  |  |  | 1.00 (Reference) |
|  | 1 | -0.158 | 0.805 | -0.197 | 0.8439 | 0.85 (0.16 - 4.18) |
| Differentiation degree | 1 |  |  |  |  | 1.00 (Reference) |
|  | 2 | -16.504 | 1385.378 | -0.012 | 0.9905 | NA (Not Calculable) |
|  | 3 | 0.063 | 1.436 | 0.044 | 0.9653 | 1.06 (0.04 - 27.71) |
| EBV DNA copy number |  | 0 | 0 | -0.096 | 0.9235 | 1.00 (1.00 - 1.00) |
| Eosinophil |  | -0.086 | 1.17 | -0.073 | 0.9418 | 0.92 (0.07 - 9.94) |
| Gingiva | 1 |  |  |  |  | 1.00 (Reference) |
|  | 2 | 0.741 | 0.774 | 0.957 | 0.3384 | 2.10 (0.47 - 10.98) |
| Probiotic intervention | Reference group |  |  |  |  | 1.00 (Reference) |
|  | ssK12 group | -0.175 | 0.593 | -0.296 | 0.7673 | 0.84 (0.26 - 2.69) |
|  | ssM18 group | -1.089 | 0.618 | -1.761 | 0.0782 | 0.34 (0.10 - 1.10) |
| Hb |  | -0.009 | 0.014 | -0.675 | 0.4994 | 0.99 (0.96 - 1.02) |
| Hypertension | 0 |  |  |  |  | 1.00 (Reference) |
|  | 1 | -0.331 | 0.593 | -0.559 | 0.5765 | 0.72 (0.21 - 2.27) |
| IFN-α |  | 1.327 | 0.517 | 2.565 | **0.0103** | 3.77 (1.56 - 11.59) |
| IFN-γ |  | -0.112 | 0.168 | -0.668 | 0.5039 | 0.89 (0.58 - 1.20) |
| IL6 |  | 0.860 | 0.216 | 3.579 | **0.0001** | 2.36 (1.63 – 3.87) |
| IL8 |  | 0.631 | 0.212 | 2.973 | **0.0029** | 1.88 (1.30 - 3.04) |
| Lips | 1 |  |  |  |  | 1.00 (Reference) |
|  | 2 | 1.638 | 1.146 | 1.429 | 0.1530 | 5.14 (0.71 - 103.64) |
| LMR |  | -0.109 | 0.143 | -0.764 | 0.4451 | 0.90 (0.65 - 1.16) |
| Lymphocyte |  | -0.657 | 0.411 | -1.596 | 0.1105 | 0.52 (0.22 - 1.13) |
| Monocyte |  | -0.787 | 1.334 | -0.59 | 0.5551 | 0.46 (0.03 - 6.04) |
| N Stages | 0 |  |  |  |  | 1.00 (Reference) |
|  | 1 | 0.288 | 1.118 | 0.257 | 0.7969 | 1.33 (0.14 - 12.95) |
|  | 2 | 0 | 0.886 | 0 | 0.9999 | 1.00 (0.16 - 6.08) |
|  | 3 | -0.56 | 0.929 | -0.602 | 0.5469 | 0.57 (0.09 - 3.73) |
| Neutrophil |  | -0.015 | 0.122 | -0.126 | 0.8994 | 0.98 (0.77 - 1.25) |
| NLR |  | 0.151 | 0.183 | 0.826 | 0.4088 | 1.16 (0.81 - 1.69) |
| NRS2002 | 1 |  |  |  |  | 1.00 (Reference) |
|  | 2 | 0.913 | 0.551 | 1.657 | 0.0976 | 2.49 (0.86 - 7.54) |
|  | 3 | 1.567 | 0.909 | 1.723 | 0.0849 | 4.79 (0.89 - 37.11) |
|  | 4 | 0.651 | 1.062 | 0.613 | 0.5400 | 1.92 (0.21 - 17.66) |
| Number of IC cycles | 2 |  |  |  |  | 1.00 (Reference) |
|  | 3 | 1.109 | 0.661 | 1.679 | 0.0932 | 3.03 (0.87 - 12.34) |
|  | 4 | -16.268 | 1696.734 | -0.010 | 0.9924 | NA (Not Calculable) |
| D50 of ORC |  | 0.028 | 0.016 | 1.717 | 0.0859 | 1.03 (1.00 - 1.07) |
| D35 of PG_average |  | -0.006 | 0.016 | -0.373 | 0.7088 | 0.99 (0.96 - 1.03) |
| Platelet |  | 0.004 | 0.004 | 1.031 | 0.3025 | 1.00 (1.00 - 1.01) |
| PLR |  | 0.007 | 0.004 | 1.804 | 0.0712 | 1.01 (1.00 - 1.02) |
| PNI |  | -0.17 | 0.067 | -2.559 | **0.0105** | 0.84 (0.73 - 0.95) |
| Saliva | 1 |  |  |  |  | 1.00 (Reference) |
|  | 2 | 0.962 | 0.623 | 1.546 | 0.1222 | 2.62 (0.79 - 9.53) |
|  | 3 | 15.941 | 1455.398 | 0.011 | 0.9913 | NA (Not Calculable) |
| SCC |  | -0.11 | 0.177 | -0.619 | 0.5356 | 0.90 (0.59 - 1.25) |
| Sex | 0 |  |  |  |  | 1.00 (Reference) |
|  | 1 | -0.347 | 0.525 | -0.66 | 0.5091 | 0.71 (0.25 - 1.98) |
| SII |  | 0.001 | 0.001 | 1.201 | 0.2299 | 1.00 (1.00 - 1.00) |
| SIRI |  | 0.19 | 0.263 | 0.722 | 0.4701 | 1.21 (0.72 - 2.09) |
| Heavy smoking | 0 |  |  |  |  | 1.00 (Reference) |
|  | 1 | 0.499 | 0.552 | 0.904 | 0.3658 | 1.65 (0.56 - 4.99) |
| Teeth (BOAS) | 1 |  |  |  |  | 1.00 (Reference) |
|  | 2 | 2.39 | 0.73 | 3.274 | 0.0011 | 10.91 (2.90 - 54.59) |
|  | 3 | 4.025 | 0.973 | 4.138 | <0.001 | 56.00 (10.13 - 509.33) |
| TNF |  | 0.449 | 0.256 | 1.753 | 0.0796 | 1.57 (1.01 - 2.74) |
| TOMO | 0 |  |  |  |  | 1.00 (Reference) |
|  | 1 | -0.734 | 0.504 | -1.458 | 0.1448 | 0.48 (0.17 - 1.27) |
| Total Weight Lost |  | 0.091 | 0.099 | 0.92 | 0.3578 | 1.09 (0.90 - 1.34) |
| T Stage | 1 |  |  |  |  | 1.00 (Reference) |
|  | 2 | 16.084 | 1495.296 | 0.011 | 0.9914 | NA (Not Calculable) |
|  | 3 | 18.665 | 1495.296 | 0.012 | 0.99 | NA (Not Calculable) |
|  | 4 | 18.952 | 1495.296 | 0.013 | 0.9899 | NA (Not Calculable) |

S Table 11. Multicollinearity diagnostics of candidate variables for the multivariable model

| Variables | GVIF^(1/(2*Df)) |
| --- | --- |
| BOAS | 2.11 |
| Teeth | 1.52 |
| IL-6 | 1.76 |
| IL-8 | 1.57 |
| IFN-α | 1.38 |
| PNI | 2.16 |
| Weekly standard drinks | 1.10 |
| Albumin | 1.67 |
| PLR | 1.64 |
| TNF | 1.37 |
| NRS | 1.12 |
| D50 of ORC | 1.10 |
| Number of IC cycles | 1.10 |

IL-6, interleukin-6; IL-8, interleukin-8; IFN-α, interferon alpha; PNI, prognostic nutritional index; PLR, platelet-to-lymphocyte ratio; TNF, tumor necrosis factor; NRS, nutritional risk screening; TNF: Tumor Necrosis Factor; ORC, oral rinsing count; CI: Confidence Interval.S Table 12. Multivariate analysis of factors associated with 'Rapid-Onset, Severe' group.

| Variables | Level | *P* | OR (95%CI) |
| --- | --- | --- | --- |
| Log-transformed IL-6 |  | **0.020** | 4.20 (1.27 – 13.86) |
| BOAS |  | **0.044** | 3.06 (1.11 – 8.44) |
| Log-transformed weekly standard drinks |  | 0.268 | 1.56 (0.77 – 3.17) |
| NRS 2002 | 1 |  | 1.00 (Reference) |
|  | 2 | 0.593 | 1.61 (0.34 – 7.57) |
|  | 3 | 0.283 | 0.23 (0.02 – 2.24) |
|  | 4 | 0.431 | 0.15 (0.01 – 4.42) |
| Number of IC cycles | 2 |  | 1.00 (Reference) |
|  | 3 | 0.808 | 1.27 (0.23 – 7.05) |
|  | 4 | 0.39 | 6.59 (0.21 – NA) |
| PNI |  | 0.393 | 0.85 (0.62 –1.18) |
| TNF |  | 0.561 | 1.28 (0.65 – 2.55) |
| IL-8 |  | 0.691 | 0.97 (0.86 – 1.10) |
| D50 of ORC |  | 0.844 | 1.00 (0.97 – 1.03) |
| Albumin |  | 0.871 | 1.04 (0.72 – 1.50) |
| IFN-α |  | 0.919 | 0.95 (0.42 – 2.17) |
| PLR |  | 0.947 | 1.00 (0.99 – 1.01) |

S Table 13. Univariate Cox regression of radiotherapy sessions to OM onset incorporating BOAS subcomponents and covariates.

| Variables | Level | β | SE | Z | *P* | HR (95%CI) |
| --- | --- | --- | --- | --- | --- | --- |
| Probiotic intervention | Reference |  |  |  |  | 1.00 (Reference) |
|  | SsK12 | -0.08 | 0.30 | -0.25 | 0.801 | 0.93 (0.52 - 1.67) |
|  | SsM18 | -0.67 | 0.31 | -2.18 | **0.030** | 0.51 (0.28 - 0.94) |
| NRS 2002 | 1 |  |  |  |  | 1.00 (Reference) |
|  | 2 | 0.57 | 0.27 | 2.08 | **0.037** | 1.77 (1.03 - 3.04) |
|  | 3 | 0.57 | 0.42 | 1.35 | 0.176 | 1.77 (0.77 - 4.04) |
|  | 4 | 0.52 | 0.53 | 0.97 | 0.333 | 1.68 (0.59 - 4.77) |
| Number of IC cycles | 2 |  |  |  |  | 1.00 (Reference) |
|  | 3 | 0.45 | 0.31 | 1.44 | 0.151 | 1.57 (0.85 - 2.90) |
|  | 4 | -1.40 | 1.01 | -1.39 | 0.166 | 0.25 (0.03 - 1.79) |
| Lips (BOAS) | 1 |  |  |  |  | 1.00 (Reference) |
|  | 2 | 0.10 | 0.47 | 0.21 | 0.833 | 1.10 (0.44 - 2.78) |
| Gingiva (BOAS) | 1 |  |  |  |  | 1.00 (Reference) |
|  | 2 | 0.26 | 0.38 | 0.68 | 0.495 | 1.30 (0.62 - 2.73) |
| Tongue (BOAS) | 1 |  |  |  |  | 1.00 (Reference) |
|  | 2 | 2.41 | 1.08 | 2.23 | **0.026** | 11.09 (1.34 - 92.15) |
| Teeth (BOAS) | 1 |  |  |  |  | 1.00 (Reference) |
|  | 2 | 0.38 | 0.29 | 1.32 | 0.188 | 1.46 (0.83 - 2.56) |
|  | 3 | 1.20 | 0.32 | 3.73 | **<0.001** | 3.33 (1.77 - 6.28) |
| Saliva (BOAS) | 1 |  |  |  |  | 1.00 (Reference) |
|  | 2 | 0.82 | 0.32 | 2.60 | **0.009** | 2.28 (1.23 - 4.23) |
|  | 3 | 2.62 | 1.09 | 2.42 | **0.016** | 13.77 (1.64 - 115.60) |
| PLR |  | 0.00 | 0.00 | 0.97 | 0.331 | 1.00 (1.00 - 1.00) |
| D50 of ORC |  | 0.00 | 0.01 | 0.73 | 0.466 | 1.00 (0.99 - 1.02) |
| IL-6 |  | 0.09 | 0.02 | 5.29 | **<0.001** | 1.10 (1.06 - 1.14) |
| IL-8 |  | 0.09 | 0.02 | 4.92 | **<0.001** | 1.09 (1.05 - 1.13) |
| TNF |  | 0.05 | 0.10 | 0.52 | 0.603 | 1.05 (0.86 - 1.29) |
| Weekly standard drinks |  | 0.02 | 0.01 | 1.94 | 0.052 | 1.02 (1.00 - 1.04) |
| PNI |  | -0.02 | 0.03 | -0.75 | 0.455 | 0.98 (0.92 - 1.04) |
| Albumin |  | -0.03 | 0.05 | -0.57 | 0.570 | 0.97 (0.89 - 1.07) |

S Table 14. Multivariable Cox regression of radiotherapy sessions to oral mucositis onset incorporating BOAS subcomponents and covariates.

| Variables | Level | β | SE | Z | P | HR (95%CI) |
| --- | --- | --- | --- | --- | --- | --- |
| Teeth (BOAS) | 1 |  |  |  |  | 1.00 (Reference) |
|  | 2 | 0.11 | 0.36 | 0.3 | 0.761 | 1.12 (0.55–2.28) |
|  | 3 | 0.92 | 0.45 | 2.04 | **0.042** | 2.50 (1.03–6.03) |
| Gingiva (BOAS) | 1 |  |  |  |  | 1.00 (Reference) |
|  | 2 | -0.24 | 0.48 | -0.49 | 0.623 | 0.79 (0.30–2.04) |
| Lips (BOAS) | 1 |  |  |  |  | 1.00 (Reference) |
|  | 2 | -0.05 | 0.65 | -0.08 | 0.937 | 0.95 (0.27–3.38) |
| Tongue (BOAS) | 1 |  |  |  |  | 1.00 (Reference) |
|  | 2 | 3.07 | 1.4 | 2.19 | **0.029** | 21.49 (1.38–335.96) |
| Saliva (BOAS) | 1 |  |  |  |  | 1.00 (Reference) |
|  | 2 | 0.8 | 0.45 | 1.78 | 0.075 | 2.23 (0.92–5.38) |
|  | 3 | 1.72 | 1.48 | 1.16 | 0.245 | 5.58 (0.31–101.31) |
| IL-6 |  | 0.05 | 0.03 | 1.91 | 0.056 | 1.05 (1.00–1.11) |
| IL-8 |  | 0.04 | 0.03 | 1.52 | 0.129 | 1.04 (0.99–1.10) |
| PNI |  | 0.07 | 0.06 | 1.14 | 0.256 | 1.07 (0.95–1.22) |
| IFN-α |  | 0.21 | 0.16 | 1.34 | 0.181 | 1.24 (0.90–1.70) |
| Weekly standard drinks |  | 0.02 | 0.01 | 1.86 | 0.062 | 1.02 (1.00–1.05) |
| Albumin |  | -0.04 | 0.08 | -0.46 | 0.645 | 0.97 (0.83–1.12) |
| PLR |  | 0 | 0 | 0.94 | 0.349 | 1.00 (1.00–1.01) |
| D50 of ORC |  | 0 | 0.01 | -0.5 | 0.614 | 1.00 (0.98–1.01) |
| NRS2002 | 1 |  |  |  |  | 1.00 (Reference) |
|  | 2 | 0.07 | 0.36 | 0.18 | 0.855 | 1.07 (0.52–2.18) |
|  | 3 | 0.04 | 0.52 | 0.08 | 0.935 | 1.04 (0.37–2.92) |
|  | 4 | -0.11 | 0.64 | -0.16 | 0.869 | 0.90 (0.25–3.18) |
| Number of IC cycles | 2 |  |  |  |  | 1.00 (Reference) |
|  | 3 | 0.41 | 0.46 | 0.9 | 0.368 | 1.51 (0.61–3.74) |
|  | 4 | -1.06 | 1.05 | -1.01 | 0.313 | 0.35 (0.04–2.72) |

**Cross-reference table of nouns**

OM: Oral Mucositis;

CTCAE: Common Terminology Criteria for Adverse Events;

RTOG: Radiation Therapy Oncology Group;

NRS2002: Nutritional Risk Screening 2002;

BOAS: Beck Oral Assessment Scale;

TPN: Total Parenteral Nutrition;

IV: Intravenous;

Wt = weight;

COPD = Chronic Obstructive Pulmonary Disease;

BOAS: Beck Oral Assessment Scale;

WHO Grade: WHO oral toxicity score;

Wk: weekend;

SFR: Salivary flow rate;

BMI: Body mass index;

EB: Epstein-Barr virus;

CYFRA21-1: Cytokeratin 19 fragment antigen 21-1;

CEA: Carcinoembryonic antigen;

SCC: Squamous cell carcinoma antigen;

IC: Induction chemotherapy;

IL: Interleukin;

IFN: Interferon;

TNF: Tumor Necrosis Factor;

NLR: Neutrophil-to-lymphocyte ratio;

PLR: Platelet-to-lymphocyte ratio;

LMR: Lymphocyte-to-monocyte ratio;

SII: Systemic inflammatory index;

SIRI: Systemic inflammatory response index;

PNI: Prognostic nutritional index;

OR: Odds Ratio;

CI: Confidence Interval;

RERI: Relative excess risk due to interaction;

AP: Attributable proportion;

SI: synergy index;

ORC, oral rinsing count.
